# Aflibercept With vs Without Reduced-Fluence Photodynamic Therapy for Polypoidal Choroidal Vasculopathy: A Randomized Clinical Trial

**DOI:** 10.1001/jamaophthalmol.2025.0250

**Published:** 2025-03-27

**Authors:** Yu Jeat Chong, Kelvin Yi Chong Teo, Wendy Wong, Anna C. S. Tan, Xinyi Su, Noa Gilead, Hiok Hong Chan, Farah Ibrahim, Beau Fenner, Charles Ong, Christopher Sun, Shaun Sim, Caroline Chee, Usha Chakravarthy, Chui Ming Gemmy Cheung

**Affiliations:** 1 Singapore Eye Research Institute, Singapore National Eye Centre, Singapore; 2 Department of Medical Retina, Singapore National Eye Centre, Singapore; 3 Department of Ophthalmology, National University Hospital, Singapore; 4 Ophthalmology and Visual Sciences Academic Clinical Program, Duke-NUS medical School, Singapore; 5 Queens University of Belfast, Belfast, Northern Ireland

## Abstract

**IMPORTANCE:**

The potential benefit of adding photodynamic therapy (PDT) to intravitreal aflibercept injection (IAI) in eyes with polypoidal choroidal vasculopathy (PCV) remains unclear.

**OBJECTIVE:**

To compare the functional and anatomical benefit of combination therapy using reduced-fluence PDT (RF-PDT) plus IAI vs IAI monotherapy in participants with PCV.

**DESIGN, SETTING, AND PARTICIPANTS:**

This double-masked, sham-controlled, randomized clinical trial was conducted at 2 centers in Singapore from January 2021 to June 2024 for participants aged 50 years or older with symptomatic macular PCV confirmed on indocyanine green angiography. Data were analyzed from January 2021 to June 2024.

**INTERVENTIONS:**

Randomization 1:1 to RF-PDT plus 2 mg of IAI or sham-PDT plus 2 mg of IAI at week 0. Follow-up was at 4 weeks and retreatment with IAI, per protocol pro re nata regimen.

**MAIN OUTCOMES AND MEASURES:**

The primary outcome was the mean change in best-corrected visual acuity (BCVA) from baseline to week 52. Secondary outcomes, not adjusted for multiple analyses, included proportion of eyes with polypoidal lesion (PL) closure at week 12 per indocyanine green angiography .

**RESULTS:**

Only 60 (43 male [71.6%] and 17 female [28.4%]; mean [SD] age, 71.3 [5.7] years) of the planned 160 participants were enrolled between January 2021 and June 2023. Among these, 30 of 30 (100%) and 30 of 30 participants (100%) in combination and monotherapy groups, respectively, returned for the 52-week follow-up. Baseline BCVA letter score (approximate Snellen equivalent [SD]) was 62.0 (20/63 [10.6]) and 62.0 (20/63 [10.7]) in the combination and monotherapy arms, respectively. At week 52, mean gain in BCVA was 12.7 (combination) vs 11.9 (monotherapy) (difference = 0.8 letters; 95% CI, −3.0 to 6.0 letters; *P* = .82). At week 12, the PL closure rate occurred in 20 of 30 eyes (66.7%) vs 10 of 30 eyes (33.3%) in the combination and monotherapy arms, respectively (difference = 33.4%; 95% CI, 9.5%-57.2%; *P* = .02).

**Conclusions and Relevance:**

With less than half of the planned sample size enrolled, no superiority in BCVA outcomes for either arm was detected and the combination arm could not be shown to be not worse (not noninferior) to the monotherapy arm. While PL closure at week 12 was greater in the combination arm, secondary outcome results, which were not adjusted for multiple analyses, should be considered hypothesis generating and not associated with a clinically relevant functional outcome in this trial.

**TRIAL REGISTRATION:**

ClinicalTrials.gov Identifier: NCT03941587

## Key Points

**Question:**

Does the combination of photodynamic therapy with intravitreal aflibercept injection lead to better visual acuity (VA) outcomes than intravitreal aflibercept injection monotherapy in participants with polypoidal choroidal vasculopathy?

**Findings:**

In this randomized clinical trial, less than half of the planned sample size was enrolled but no differences in best-corrected VA outcomes were identified. Polypoidal lesion closure rates at week 12 were greater in the combination group.

**Meaning:**

These data could not determine if VA outcomes were better or worse with combination vs monotherapy for polypoidal choroidal vasculopathy, although results supported the hypothesis that combination therapy increased the chance of polypoidal lesion closure at week 12.

## Teaser

This study compares the functional and anatomical benefit of combination therapy using reduced-fluence photodynamic therapy plus intravitreal aflibercept injection vs intravitreal aflibercept injection monotherapy in participants with polypoidal choroidal vasculopathy.

## Introduction

Polypoidal choroidal vasculopathy (PCV) is a subtype of neovascular age-related macular degeneration. It accounts for up to 50% of Asian patients presenting with neovascular age-related macular degeneration, compared with 20% in Caucasian populations.^1,2,3,4^ PCV is characterized by the presence of polypoidal lesions (PL) with an associated branching neovascular network that is best visualized with indocyanine green angiography (ICGA).^5,6,7^ Vision loss in PCV may occur as a result of exudation or bleeding.^8^ As subfoveal blood and exudates are neurotoxic, this can result in scarring and macular atrophy.^9,10^ Poor visual outcomes have been reported in cases with subretinal fibrosis and submacular hemorrhage.^11,12^

As PLs are widely considered the most obvious source of hemorrhage, they have been targeted for closure with focal laser and photodynamic therapy (PDT).^13,14^ The EVEREST II trial^15^ demonstrated that combining intravitreal ranibizumab with PDT achieved superior visual gains (8.3 vs 5.1 Early Treatment Diabetic Retinopathy Study [ETDRS] letters at 12 months) and a higher rate of PL closure compared with intravitreal ranibizumab monotherapy (69.3% vs 34.7%). At 6 years follow-up, there was no difference in the change in visual acuity (VA) from baseline between the groups.^16^ In contrast, the PLANET trial^17,18^ showed that good visual outcomes are compatible with inactivated PL with the use of intravitreal aflibercept injection (IAI) monotherapy. Polyp inactivation is characterized by the absence of fluid despite the persistence of PL on ICGA. However, the complete PL closure rate on ICGA remained low in the PLANET trial, which was less than 50% in both arms.

While the PLANET trial demonstrated the efficacy and safety of intravitreal aflibercept monotherapy, there are several limitations to the study. Importantly, whether the addition of PDT confers additional benefit to intravitreal aflibercept could not be evaluated. Additionally, only 15% of the study population met the criteria for rescue PDT.^17^ Furthermore, with fixed dosing in the first year, the mean number of injections in both arms was relatively frequent at 8.1 injections. A treat-and-extend regimen was subsequently optional after the first year, which resulted in a reduced treatment burden to a mean number of 4.6 injections.^18^

Important clinical questions remain regarding optimal treatment strategy for PCV. First, whether prompt combination of intravitreal injections (IVT) and PDT at baseline could have achieved even better VA outcomes than IVT monotherapy. Second, if PDT had been given as an adjunct at baseline rather than as a rescue as in the PLANET study, whether a higher PL closure rate could have been achieved. Third, whether combination with PDT could have led to a reduced number of IVT if fixed dosing was not mandated. Lastly, the efficacy and safety of reduced fluence (RF-PDT), as opposed to standard fluence PDT, remains unclear.^19,20^

To address these questions, we conducted a multicenter randomized clinical trial comparing a single treatment of RF-PDT plus IAI with follow-up IAI vs IAI monotherapy administered in a pro re nata (PRN) regimen for the treatment of PCV.

## Methods

### Study Design

This 52-week, multicenter, randomized, double-masked clinical trial of participants with PCV was conducted at the Singapore National Eye Centre (SNEC) and the National University Hospital (ClinicalTrials.gov Identifier: NCT03941587) between January 2021 to June 2024. The study was conducted in accordance with the Declaration of Helsinki. The study protocol received approval from the institutional review boards/independent ethics committee of each the participating centers and followed the Consolidated Standards of Reporting Trials (CONSORT) reporting guidelines (eFigure 1 in Supplement 1).

### Participants and Treatments

All participants gave written informed consent prior to participating in the trial. Participants were aged 50 years or older with symptomatic macular PCV and were treatment naive in the study eye. Participants received a small stipend to cover for their travel costs. The eligible best-corrected visual acuity (BCVA) letter score in the study eye was at least 4 letters (approximately 20/800 Snellen equivalent), measured using the ETDRS VA charts at 4 m following refraction. Full inclusion and exclusion criteria are provided in the eMethods in Supplement 1. The Singapore National Eye Centre ocular reading center acted as the centralized reading center (CRC) to confirm the diagnosis of PCV prior to randomization detected on ICGA using previously published criteria.^21^

Eligible participants were randomized to their treatment arms with a blocked randomization method using a ratio of 1:1. Both participants and the investigators were masked to the treatment received. Each participant could have only 1 study eye. If both eyes were treatment naive, the eye with the worse VA was selected as the study eye. At baseline visit, participants in the combination arm received a single treatment of RF-PDT plus IAI; participants in the monotherapy arm received sham RF-PDT plus IAI. IAI was given at a dose of 2 mg/.05 mL. For participants in the combination arm, verteporfin was infused at a dose of 6 mg/m^2^ body surface area over 10 minutes. Fifteen minutes after the start of the infusion, a laser light dose of 25 J/cm^2^ with a wavelength of 689 nm was applied to an area that encompassed the entire lesion (PL and BVN), as observed on ICGA. For participants in the monotherapy arm, a sham infusion was prepared using 5% dextrose water solution. The sham-laser procedure was performed to mimic active RF-PDT with laser applied at 0 fluence to the target lesion area. All participants were instructed to avoid direct sunlight for 48 hours after.

After the baseline visit, all participants were assessed every 4 weeks until week 52. BCVA and optical coherence tomography were performed at every visit. In addition, fundus fluorescein angiography and ICGA were performed at baseline, week 12, and week 52. From week 4 to week 52, participants in either arm could receive additional IAI based on a PRN regimen, according to protocol-specific retreatment criteria (eMethods in Supplement 1). eFigure 3 in Supplement 1 summarizes the treatment regimen for the different arms. The eMethods in Supplement 1 provide further details regarding imaging protocol and CRC assessments. A typical example of PL closure at week 12 is provided in eFigure 2 in Supplement 1.

The rationale to use RF-PDT was based on potential concerns that acute macular exudation can occur shortly after treatment with full-fluence PDT.^22^ Experimental animal models have shown that halving the fluence did not modify the closure rates of choroidal neovascularization.^23^ Other animal models have also shown that the spike in choroidal neovascularization leakage after PDT is not fully abrogated by concomitant anti-vascular endothelial growth factor (VEGF) therapy.^24^ As we wished to mitigate this risk, we elected to use half-fluence PDT while maintaining the dose of 6 mg/m^2^ body surface area.

### Study Objectives

The primary outcome was the difference in the mean change in BCVA between groups at week 52. Secondary outcomes included the proportion of participants who gained or lost at least 5, 10, or 15 ETDRS letters at week 52; PL closure rate at week 12; and the difference between groups in the mean change in central subfield thickness (CST) from baseline to week 52. Other anatomic outcomes included presence of any subretinal fluid or intraretinal fluid. Additional outcomes included mean number of IVT and frequency and severity of ocular and nonocular adverse events over time. Multimodal image grading for anatomical outcomes was performed by trained graders from the CRC.

### End Points and Sample Size Calculations

Assuming a dropout of 10%, 80 participants per treatment arm were judged adequate to demonstrate noninferiority of the combination arm within a 5-letter noninferiority margin; α = .025 (1-sided); power = .80; assuming a BCVA gain of 11.5 letters in the combination arm vs 10.7 letters in the monotherapy arm. Furthermore, it was judged possible to demonstrate superiority of combination over monotherapy arms with a 3-letter superiority difference, assuming a BCVA gain of 13.7 letters in the combination arm vs 10.7 letters in the monotherapy arm (α = .025; [1-sided]; power = .80) and assuming a dropout rate of 10%. Additionally, it was concluded this sample size would detect a difference in number of injections of at least 2 based on a reduction from 7.3 in the monotherapy arm vs 5.2 in the combination arm with more than 99% power, based on injection numbers in the EVEREST II study.^15^

PL regression was the first of several secondary objectives. With considerable delays in trial initiation associated with the COVID-19 pandemic, followed by a global disruption of verteporfin supply for PDT, upon resumption of verteporfin supply in January 2022, the steering committee met and deemed the goal of recruiting 160 participants was untenable within the timeframe of our funding, which would end by July 2024.^25^ The protocol was therefore modified (Supplement 2) with approval by the study steering committee and the data safety monitoring committee, to consider the primary outcome as PL closure in an amended protocol and statistical analysis plan dated November 2022. However, following review for publication, this revision was not accepted by reviewers and editors, resulting in presentation of results per the original protocol.

### Statistical Analysis

All statistical tests were 2-sided. For the primary outcome, the level of statistical significance was .05. No other *P* values should be considered statistically significant, as there was no a priori adjustment for multiple analyses. Categorical variables were presented as the number and percentage of participants in each category and summarized using descriptive statistics. Our full analysis set included all randomized participants, since no participants dropped out of the study. The χ^2^ test was performed on the primary variable and PL closure variable. Comparisons between categorical variables also were performed with the Fisher test. For continuous data, normality of data was assessed using the Shapiro-Wilk test. For parametric data, a *t* test was used. For nonparametric data, the Mann-Whitney *U* test was used. The change in the parameters of CST, BCVA, greatest linear diameter of lesion area, and central subfield volume were analyzed using a mixed-effect model to account for baseline differences. The adjusted mean (95% CI) change was estimated for each parameter.

## Results

### Participant Disposition and Baseline Characteristics

A total of 60 participants (43 male [71.6%] and 17 female [28.4%]; mean [SD] age, 71.3 [5.7] years) were randomized to receive either combination therapy (n = 30) or IAI monotherapy (n = 30). All randomized participants completed the 52-week study. Baseline characteristics are summarized in Table 1. The mean (SD) BCVA was 62.0 (10.6) (Snellen equivalent, 20/63) and 62.0 (10.7) (Snellen equivalent, 20/63) in the combination arm and the monotherapy arm, respectively; mean (SD) greatest linear diameter of the lesion area was 2856.9 (1188.6) μm and 2819.2 (1198.9) μm, and mean (SD) CST was 358 (69.1) μm and 435 (110.0) μm in the combination arm and monotherapy arm, respectively. All lesions showed evidence of activity on either fundus fluorescein angiography or optical coherence tomography.

**Table 1.** Baseline Demographics and Clinical Characteristics Based on Randomized Population

Characteristics	No. (%)	
	Aflibercept 2.0 mg with RF-PDT (n = 30)	Aflibercept 2.0 mg with sham RF-PDT (n = 30)
Age, y, mean (SD)	70.0 (5.6)	72.6 (5.8)
Sex		
Male	19 (63.3)	24 (80.0)
Female	11 36.7)	6 (20)
Ethnicity^a^		
Chinese	28 (93.3)	26 (86.7)
Malay	1 (3.3)	3 (10.0)
Indian	1 (3.3)	1 (3.3)
BCVA letter score, mean (SD)	62.0 (10.6)	62.0 (10.7)
≥70 (20/40 or better)	9 (30.0)	10 (33.3)
<35 (worse than 20/200)	0	0
Presence of active fluorescein leakage	30 (100.0)	30 (100.0)
ICGA grading		
Presence of PL	30 (100.0)	30 (100.0)
Presence of BVN	30 (100.0)	30 (100.0)
GLD of lesion area, μm, mean (SD)	2856.9 (1188.6)	2819.2 (1198.9)
OCT grading		
CST, μm, mean (SD)	358 (69.1)	435 (110.0)
CSV, mm^3^, mean (SD)	0.32 (0.1)	0.32 (0.1)
SFCT, μm, mean (SD)	240.5 (98.6)	233.6 (97.3)
Proportion with SRF	29 (96.7)	30 (100.0)
Proportion with IRF	12 (40.0)	14 (46.7)

Abbreviations: BCVA, best corrected visual acuity; BVN, branching vascular network; CST, central subfield thickness was defined as the averaged thickness of the 1 mm area centered over the fovea, from the internal limiting membrane to the lower boundary of Bruch’s membrane; CSV, central subfield volume was defined as the retinal volume of the 1mm area centered over the fovea; GLD, greatest linear diameter; ICGA, indocyanine green angiography; IRF, intraretinal fluid; OCT, optical coherence tomography; PDT, photodynamic therapy; PL, polypoidal lesion; RF-PDT, reduced-fluence PDT; SFCT, subfoveal choroidal thickness was defined as the distance from the outer border of the retinal pigment epithelium to the inner border of the sclera; SRF, subretinal fluid.

^a^ Ethnicity was reported according to patient national identity card.

### Efficacy

#### Visual Acuity Outcomes

**Figure 1.**
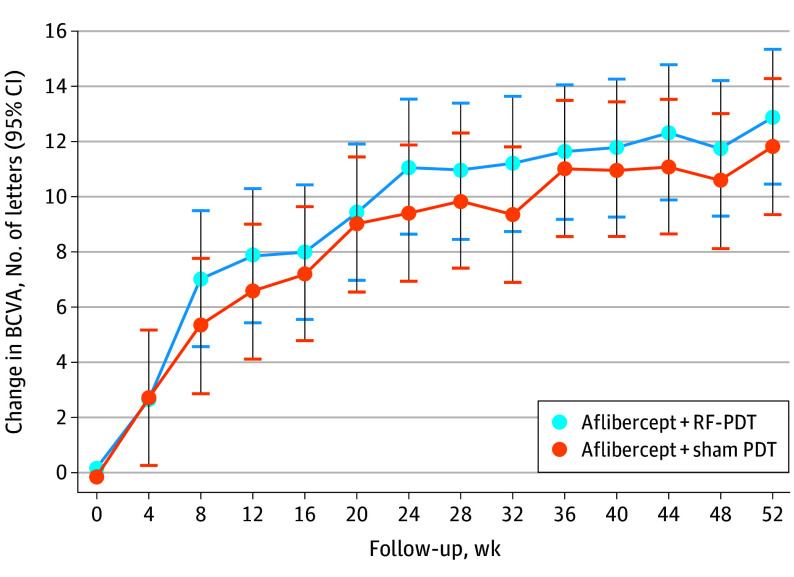
Adjusted Mean Best-Corrected Visual Acuity (BCVA) Change Between Combination and Monotherapy Arms Over 52 Weeks Vertical lines represent 95% CIs. PDT indicates photodynamic therapy; RF-PDT, reduced-fluence PDT.

The mean (SD) gain in BCVA from baseline increased further to 12.7 (7.3) letters vs 11.9 (10.3) letters at week 52 (difference = 0.8 letters; 95% CI, −3.0 to 6.0 letters; *P* = .82) in the combination arm and monotherapy arm. The model, which adjusted for baseline BCVA, estimated the mean gain at week 52 to be 12.9 and 11.8 letters, respectively, (difference = 1.1 letters; 95% CI, −2.4 to 4.5 letters; *P* = .54). Figure 1 demonstrates the adjusted mean BCVA change for the different arms.

At week 52, 33.3% of participants in both arms experienced a BCVA gain of 15 or more ETDRS letters. Neither group had any participants who lost 5 or more ETDRS letters. The proportion of eyes with BCVA letter score of 70 or more (20/40 or better) increased from 30.0% and 33.3% (difference = 0.3%; 95% CI, −26.9 to 20.2; *P* = 1.00) at baseline to 80.0% and 73.3% (difference = 6.7%; 95% CI, −14.7 to 28.0; *P* = .76) at week 52 in the combination and monotherapy groups, respectively, as summarized in Table 2.

**Table 2.** Visual and Anatomical Outcomes at Week 52

Characteristic	No. (%)		Difference (95% CI)	*P* value
	Aflibercept 2.0 mg with RF-PDT (n = 30)	Aflibercept 2.0 mg with sham RF-PDT (n = 30)		
Visual outcomes				
Final BCVA, letter score (approximate Snellen equivalent), mean (SD)	75.1 (7.5) (20/32)	73.6 (9.6) (20/32)	1.5 (−3.0 to 5.0)	.73
BCVA change, letters, mean (95% CI)^a^	12.9 (10.4-15.3)	11.8 (9.4-14.2)	1.1 (−2.4 to 4.5)	.54
Proportions with ≥15 gain	10 (33.3)	10 (33.3)	NA	NA
Proportions with ≥5 loss	0	0	NA	NA
Proportions with ≥70	24 (80.0)	22 (73.3)	6.7 (−14.7 to 28.0)	.76
Proportions with ≤35	0	0	NA	NA
Anatomical outcomes				
Presence of active fluorescein leakage	9 (30.0)	15 (50.0)	20.0 (−47.6 to 7.6)	.19
Presence of BVN	30 (100.0)	29 (96.7)	3.3 (−3.1 to 9.8)	1.00
GLD of lesion area, μm, mean (SD)	3073.0 (1361.0)	2600.0 (938.0)	473.0 (−131.1 to 1077.1)	.08
GLD of lesion area change, μm,^a^ mean (95% CI)	−157.6 (−335.8 to 20.5)	−106.5 (−284.7 to 71.6)	51.1 (−303.0 to 200.8)	1.00
CST, μm, mean (SD)	268.0 (74.4)	283.0 (53.8)	15.0 (−48.6 to 18.6)	.20
CST change, μm,^a^ mean (95% CI)	−119.8 (−141.1 to −98.4)	−123.1 (−144.8 to −101.8)	3.3 (−27.4 to 34.1)	.83
CSV, mm^3^, mean (SD)	.21 (.06)	.23 (.04)	0.02 (−0.05 to 0.01)	.09
CSV change, mm^3^,^a^ mean (95% CI)	−.08 (−.11 to −.06)	−.11 (−.14 to −.08)	0.03 (−0.01 to 0.07)	.17
SFCT, μm, mean (SD)	191.0 (99.0)	183.0 (88.6)	8.0 (−40.6 to 56.6)	.74
SFCT change, μm,^a^ mean (95% CI)	−56.7 (−106.6 to −6.8)	−44.8 (−94.7 to 5.1)	11.9 (−82.5 to 58.7)	1.00
Proportion with any SRF at last visit	10 (33.3)	10 (33.3)	NA	NA
Proportion with any IRF at last visit	3 (10.0)	4 (13.3)	3.3 (−19.6 to 12.9)	1.00
Proportion of eyes with PL closure	22 (73.3)	14 (46.7)	26.6 (2.8 to 50.5)	.07
Proportion with PED at last visit	21 (70)	23 (76.7)	6.7 (−28.9 to 15.6)	.77

Abbreviations: BCVA, best-corrected visual acuity; BVN, branching vascular network; CST, central subfield thickness was defined as the averaged thickness of the 1 mm area centered over the fovea, from the internal limiting membrane to the lower boundary of Bruch membrane; CSV, central subfield volume was defined as the retinal volume of the 1mm area centered over the fovea; GLD, greatest linear diameter; IRF, intraretinal fluid; NA, not applicable; PDT, photodynamic therapy; PED, pigment epithelial detachment; PL, polypoidal lesion; RF-PDT, reduced-fluence PDT; SFCT, subfoveal choroidal thickness was defined as the distance from the outer border of the retinal pigment epithelium to the inner border of the sclera; SRF, subretinal fluid.

^a^ Adjusted for baseline difference.

#### Polypoidal Lesion Closure at Week 12

**Figure 2.**
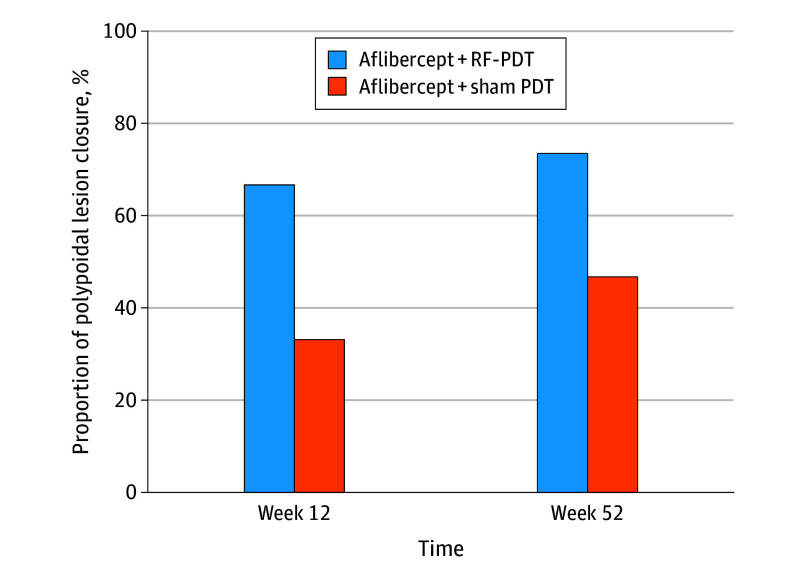
Proportion of Polypoidal Lesion Closure at Week 12 and Week 52 Between the Treatment Arms PDT indicates photodynamic therapy; RF-PDT, reduced-fluence PDT.

At week 12, 20 of 30 participants in the combination arm (66.7%) vs 10 of 30 participants in the monotherapy arm (33.3%) achieved PL closure in the study eye (difference = 33.4%; 95% CI, 9.5-57.2; *P* = .02). At week 52, the proportion of study eyes with PL closure increased to 22 of 30 participants (73.3%) and 14 of 30 participants (46.7%) for the combination and monotherapy arms, respectively (difference = 26.6%; 95% CI, 2.8-50.5; *P* = .07). These results are summarized in Figure 2.

**Figure 3.**
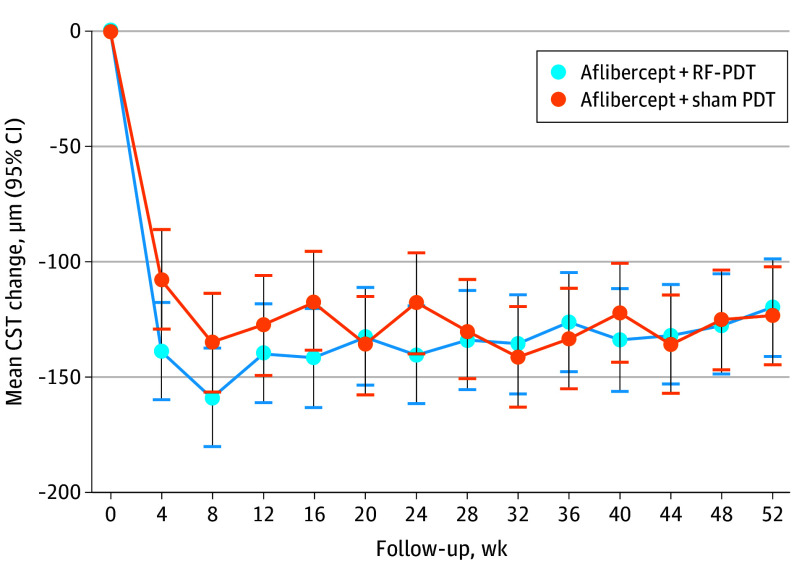
Adjusted Mean Central Subfield Thickness (CST) Change Between Combination and Monotherapy Arms Over 52 Weeks Vertical lines represent 95% CIs. PDT indicates photodynamic therapy; RF-PDT, reduced-fluence PDT.

At week 12, the adjusted mean change in CST was −139.3 μm and −127.1 μm for the combination and monotherapy arm, respectively (difference = 12.2 μm; 95% CI, −43.0 to 18.5 μm; *P* = .43). These outcomes at week 52 were −119.8 μm and −123.1 μm (difference = 3.3 μm; 95% CI, −27.4 to 34.1 μm; *P* = .83), respectively. At week 52, there was a reduction in central subfield volume in both treatment arms, −0.08 mm^3^ vs −0.11 mm^3^ (difference = 0.03 mm^3^; 95% CI, −0.01 to 0.07 mm^3^; *P* = .17). Figure 3 demonstrates the adjusted mean change in CST for the different arms. Other anatomical outcomes are summarized in Table 2.

#### Treatment Exposure

At week 52, the mean (SD) number of intravitreal aflibercept injections was 4.0 (2.0) and 4.9 (1.7) in the combination and monotherapy arms, respectively, (difference = 0.9; 95% CI, −0.06 to 1.86; *P* = .06). Also, 1 of 30 participants (combination) (3.3%) and 2 of 30 participants (monotherapy) (6.7%) required a total of 8 or more injections by week 52 (difference = 3.4%; 95% CI, −14.3 to 7.7; *P* = 1.00) and 3 of 30 (combination) (10.0%) and 10 of 30 (monotherapy) (33.3%) required 3 initial consecutive doses of 4 weekly IAI (difference = 23.3%; 95% CI, −43.3 to −3.3; *P* = .23).

### Safety

Neither treatment group experienced cases of endophthalmitis, retinal detachment, or treatment-emergent vitreous or submacular hemorrhage. There were no adverse events deemed related to the study. Further information is detailed in eTable 1 in Supplement 1.

## Discussion

We report the results of a randomized clinical trial that compares the efficacy of combination therapy, a single treatment of RF-PDT plus IAI at baseline with follow-up IAI vs IAI monotherapy for participants with PCV. Our key findings are as follows: (1) enrollment could not be completed in a feasible time, limiting confidence in the findings and increasing the chance of type 1 and type 2 errors; (2) neither arm had superior visual acuity outcomes compared with the other; (3) in association with less than half of the planned sample size enrolled, the combination arm could not be shown to be not worse (not noninferior) to the monotherapy arm; (4) the combination arm may have had superior closure rate of PL at week 12 compared with IAI monotherapy, although the statistical analysis was not adjusted for multiplicity; and (5) a difference in the number of IVT using a PRN regimen could not be identified.

For practical and safety reasons, PDT was performed first, followed by IAI. Application of PDT requires the placement of a contact lens after which laser exposure is made.^26^ Owing to the fact that light activation of the drug occurs, visualization of the region to be lasered is under extremely low-illumination conditions, which can be challenging if the surface and intraocular media are disturbed by prior administration of sterilizing iodine solution and or intravitreal drug floating within the vitreous compartment. Also, there is the theoretical risk of bacterial contamination if a contact lens is placed on a recently injected eye.

Of note, we elected to perform PDT only once at baseline in the combination group. It is unknown if more PDT sessions would have resulted in better, no different, or worse outcomes. This decision was driven by concern over potential choriocapillaris damage and risk of macular atrophy with repeated PDT sessions.^27,28^ This decision was also influenced by the global shortage of verteporfin supply.

The combined treatment arm received a reduced fluence laser exposure of 25 J, unlike the original PDT protocols, in which full fluence of 50 J was used.^21,29^ This also differs from the EVEREST II study that applied the laser at full fluence.^30^ As we did not compare RF-PDT vs full-fluence PDT, it is not possible to know what the differential effect might have been on polyp closure. Of note, the polypoidal closure rate was 73.3% among 30 participants in our combination group at week 52 compared with 69.3% among 168 participants in the EVEREST II study that used full-fluence PDT.^30^ Our data cannot determine if polypoidal closure rates are affected adversely by reduced fluence.

Comparison between the current study and the EVEREST II and PLANET studies, including retreatment criteria and type of anti-VEGF given, are summarized in eTable 2 in Supplement 1. While there are differences in terms of anti-VEGF agents used in this trial and EVEREST II (ranibizumab), our group previously demonstrated that aflibercept monotherapy with a mean of 8 injections achieved a week 52 PL closure rate between 41.6% to 55.2%.^31^ It is possible that the higher week 52 PL closure rate of 73.3% in this study could be attributable to the addition of PDT.

### Strengths and Limitations

The current study has several strengths. First it was a prospectively designed randomized clinical trial with comprehensive imaging and phenotyping of participants. There were several novel aspects of the study design that has specifically addressed knowledge gaps.

There are limitations to this study. First, delays in recruitment due to the COVID-19 pandemic and the global shortage of verteporfin led to the decision to reduce the total number of participants.^25,32^ Second, visual and anatomical benefits in the longer term beyond 52 weeks were not assessed.

## Conclusions

In summary, with less than half of the planned sample size enrolled, no superiority in BCVA outcomes for either arm was detected and the combination arm could not be shown to be not worse (not noninferior) to the monotherapy arm. While PL closure at week 12 was greater in the combination arm, secondary outcome results, which were not adjusted for multiple analyses, should be considered hypothesis generating and not associated with a clinically relevant functional outcome in this trial. The potential ability to close the PL more effectively with IAI combined with PDT compared with IAI monotherapy would suggest a potential role for combination therapy in eyes, wherein the treating physician judges that monotherapy is resulting in an inadequate response, although further studies would be needed to confirm this hypothesis.

## Notes

Supplement 1. eMethods. eFigure 1. Consort diagram eFigure 2. Typical example of polypoidal choroidal vasculopathy lesion closure on indocyanine green angiography. eFigure 3. Treatment regimen of the combination and monotherapy arms eTable 1. Details of non-ocular adverse events eTable 2. Comparison of study design and results between the EVEREST II (year 1), PLANET (year 1) and the current study.

Supplement 2. Amended trial protocol

Supplement 3. Data sharing statement
