# Supplementary material for: Aflibercept With vs Without Reduced-Fluence Photodynamic Therapy for Polypoidal Choroidal Vasculopathy: A Randomized Clinical Trial
Source: JAMA Ophthalmol. 2025 Mar 27;143(5):393–9. doi: 10.1001/jamaophthalmol.2025.0250 (PMC11950976; doi:10.1001/jamaophthalmol.2025.0250)
Supplement: Supplement 1. — eMethods. eFigure 1. Consort diagram eFigure 2. Typical example of polypoidal choroidal vasculopathy lesion closure on indocyanine green angiography. eFigure 3. Treatment regimen of the combination and monotherapy arms eTable 1. Details of non-ocular adverse events eTable 2. Comparison of study design and results between the EVEREST II (year 1), PLANET (year 1) and the current study. [file jamaophthalmol-e250250-s001.pdf]

Supplemental Online Content

Chong YJ, Chong KY, Wong W, et al. Aflibercept with vs without reduced-fluence photodynamic therapy for polypoidal choroidal vasculopathy. *JAMA Ophthalmol*. Published online March 20, 2025. doi:10.1001/jamaophthalmol.2025.0250

eMethods.

eFigure 1. Consort diagram

eFigure 2. Typical example of polypoidal choroidal vasculopathy lesion closure on indocyanine green angiography

eFigure 3. Treatment regimen of the combination and monotherapy arms

eTable 1. Details of non-ocular adverse events

eTable 2. Comparison of study design and results between the EVEREST II (year 1), PLANET (year 1) and the current study

This supplemental material has been provided by the authors to give readers additional information about their work.

© 2025 Chong YJ et al. *JAMA Ophthalmology*.

eMethods

Patient inclusion and exclusion criteria

Inclusion criteria

1. Participants aged >= 50 years old at the time of informed consent.

2. Provide written informed consent.

3. Willingness and ability to comply with all scheduled visits and study procedures.

4. Confirmed diagnosis of symptomatic macular PCV based on ICGA.

5. Activity of PCV confirmed by exudative activity involving the macula on OCT or FA or both.

   a) Presence of intra retinal or subretinal fluid/blood as seen on OCT

   b) Treatment naïve

*NO previous treatment with intravitreal anti-VEGF agents, regardless of the indication

*NO previous thermal laser in the macular region, or verteporfin photodynamic therapy (vPDT), regardless of indication

*NO other previous treatment for nAMD, except oral supplements and traditional Chinese medicine

6. An ETDRS BCVA of at least 4 letters (Snellen equivalent approximately 20/800 or better) in the study eye.

7. Greatest Linear Dimension (GLD) of the total lesion area (BVN + polyps) < 5400µm (~9 MPS Disc Areas) as delineated by ICGA.

Exclusion criteria

1. Medical condition that, in the opinion of the investigator, would preclude participation in the study (e.g. unstable medical status including blood pressure, cardiovascular disease, and glycaemic control).
2. Participation in an investigational trial within 30 days of enrolment which involves treatment with unapproved investigational drug.

© 2025 Chong YJ et al. *JAMA Ophthalmology*.

3. Known allergy to any component of the study drug.
4. Blood pressure > 180/110 (systolic above 180 OR diastolic above 110 on repeated measurements). If blood pressure is brought below 180/110 by anti-hypertensive treatment, individual can become eligible.
5. Myocardial infarction, other acute cardiac event requiring hospitalization, stroke, transient ischemic attack, or treatment for acute congestive heart failure within 4 months prior to randomisation.
6. Systemic anti-VEGF or pro-VEGF treatment within four months prior to randomization or anticipated use during the study.
7. Amblyopia or blind in one eye.

Study eye

1. Eye with intraretinal or subretinal fluid due to causes other than PCV.
2. An ocular condition is present (other than PCV) that, in the opinion of the investigator, might affect intra or sub retinal fluid or alter visual acuity during the course of the study (e.g., DME, vein occlusion, uveitis or other ocular inflammatory disease, neovascular glaucoma, etc.).
3. Substantial cataract that, in the opinion of the investigator, is likely to be decreasing visual acuity by more than three lines (i.e., cataract would be reducing acuity to worse than 20/40 if eye was otherwise normal).
4. Any intraocular surgery within 1 month of enrolment.
5. Treatment with intravitreal corticosteroids.
6. History of retinal detachment or surgery for retinal detachment.
7. History of vitrectomy.
8. History of macular hole.
9. Evidence of vitreomacular traction that may preclude resolution of macular oedema > 4-disc areas of intra/sub retinal hemorrhage.
10. Aphakia.

© 2025 Chong YJ et al. *JAMA Ophthalmology*.

11. Exam evidence of external ocular infection, including conjunctivitis, chalazion, or significant blepharitis.

Other eye

1. Active intraocular inflammation.
2. History of uveitis.

Re-treatment criteria

1. Loss of BCVA ≥ 5 letters
2. Presence of any IRF or SRF from week 4 to week 12
3. From weeks 16 onwards, a more relaxed retreatment criteria set was applied: presence of ≥200um of SRF, or an increase in IRF or SRF compared to previous visit; the presence of new retinal hemorrhage

© 2025 Chong YJ et al. *JAMA Ophthalmology*.

eFigure 1.

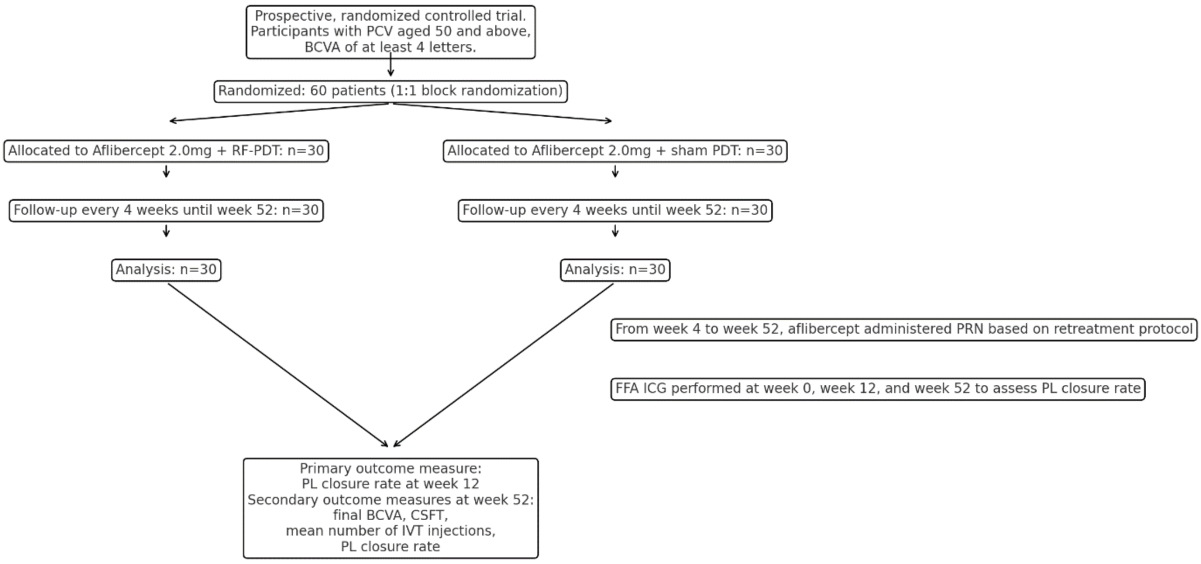

The disposition of study participants and randomisation into the combination arm with Aflibercept+RF-PDT, and monotherapy arm with Aflibercept+Sham-PDT

© 2025 Chong YJ et al. *JAMA Ophthalmology*.

eFigure 2.

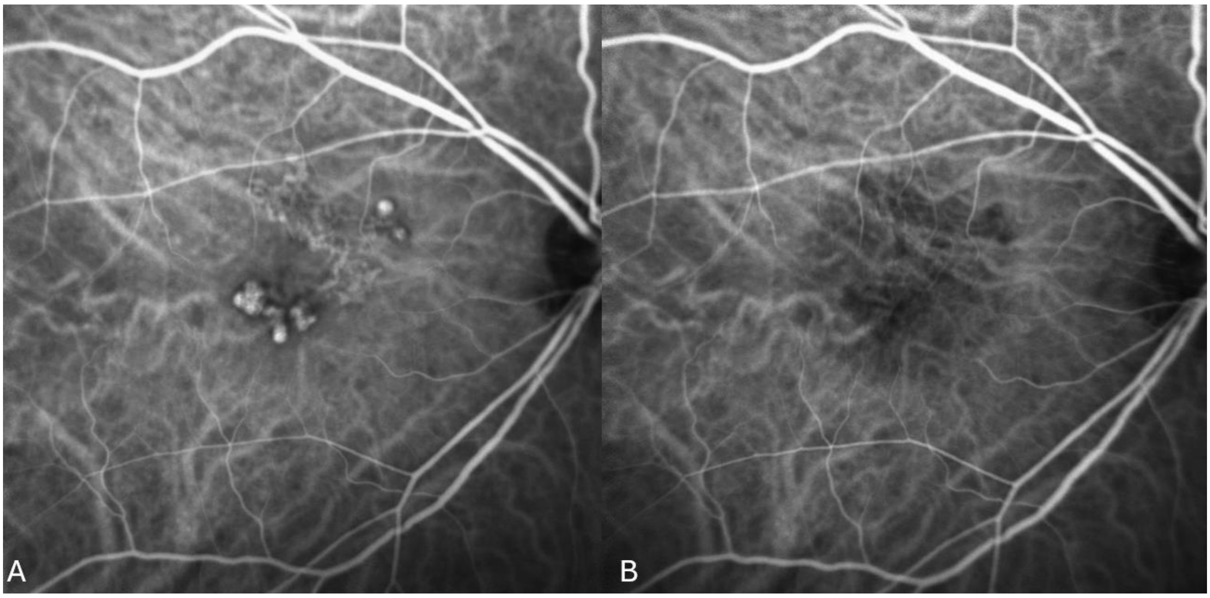

A: Indocyanine green angiography (ICGA) demonstrating polypoidal lesions (PL) at baseline;

B ICGA at week 12 with PL closure

© 2025 Chong YJ et al. *JAMA Ophthalmology*.

eFigure 3.

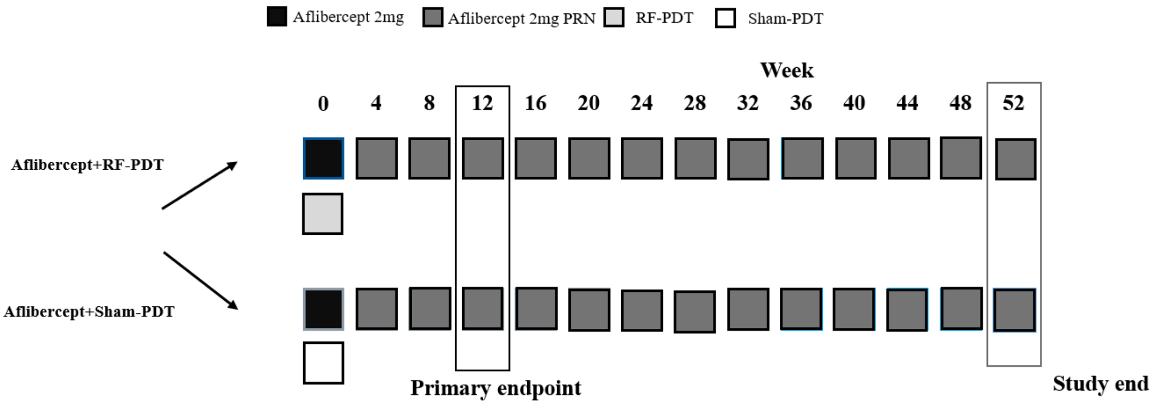

Treatment regimen of the combination and monotherapy arms

© 2025 Chong YJ et al. *JAMA Ophthalmology*.

eTable 1

Adverse Event	Category	Relation to Study
Urge incontinence*	Non-ocular adverse event	Unrelated
Overactive bladder*	Non-ocular adverse event	Unrelated
Nipple discharge*	Non-ocular adverse event	Unrelated
Occasional chest tightness*	Non-ocular adverse event	Unrelated
Left breast benign intraductal papilloma*	Non-ocular adverse event	Unrelated
Compression fracture from a fall	Serious adverse event	Unrelated
Parathyroidectomy	Serious adverse event	Unrelated
Hospitalization secondary to electrolyte derangements	Serious adverse event	Unrelated
Suspected gout flare	Serious adverse event	Unrelated
Chest tightness with subsequent PCI (Percutaneous Coronary Intervention)	Serious adverse event	Unrelated

Details of non-ocular adverse events. *All of these events occurred in the same patient.

© 2025 Chong YJ et al. *JAMA Ophthalmology*.

eTable 2.

	**EVEREST II (Year 1)**	**PLANET (Year 1)**	**Current study**
**Study Design**			
Anti-VEGF agent	Ranibizumab	Aflibercept	Aflibercept
Anti-VEGF regime	X 3 monthly then PRN	X 3 monthly then 8 weekly	PRN
Timing of first PDT	Baseline	Prohibited within	Baseline
Repeat PDT	Repeatable with minimum interval of 90 days	Repeatable with minimum interval of 90 days	No repeat
PDT settings	Full fluence	Full fluence	Half fluence
Protocol specific retreatment criteria	BCVA loss due to disease activity seen on OCT	No specific retreatment criteria but rather rescue therapy if meets BCVA based criteria below with evidence of PL on ICGA	Tolerate SRF <200um from week 16 onwards
BCVA-based criteria for PDT	No BCVA based criteria	≤73 ETDRS letters with BCVA change of <5 ETDRS letters OR BCVA gain ≥ 5 but ≤10 ETDRS letters and PDT deemed beneficial	No BCVA based criteria
Baseline BCVA (letters)	Combination: 61.1<br>Mono: 61.2	Combination: 57.7<br>Mono: 59.0	Combination: 62.0<br>Mono: 62.0
**Key results**			
Number of IVT required by week 52 (mean)	Combination:4.0 (median)<br>Mono:7.0 (median)	Both arms: 8.1	Combination: 4.0<br>Mono: 4.9
Polyp closure at week 52 (%)	Combination: 69.3<br>Mono: 34.7	Combination: 45.0<br>Mono: 39.0	Combination:73.3<br>Mono:46.7
BCVA gain at week 52 (letters)	Combination: 8.3<br>Mono:5.1	Combination:10.8<br>Mono:10.7	Combination: 12.7<br>Mono: 11.9

PDT, photodynamic therapy BCVA, best corrected visual acuity **ETDRS**, Early Treatment Diabetic Retinopathy Study

© 2025 Chong YJ et al. *JAMA Ophthalmology*.
