# Supplementary material for: Aflibercept With vs Without Reduced-Fluence Photodynamic Therapy for Polypoidal Choroidal Vasculopathy: A Randomized Clinical Trial
Source: JAMA Ophthalmol. 2025 Mar 27;143(5):393–9. doi: 10.1001/jamaophthalmol.2025.0250 (PMC11950976; doi:10.1001/jamaophthalmol.2025.0250)
Supplement: Supplement 2. — Amended trial protocol [file jamaophthalmol-e250250-s002.pdf]

Restricted. Sensitive (Normal)

SUPERSEDED

SingHealth

CLINICAL TRIAL PROTOCOL

PROTOCOL TITLE:
A multi-center, randomized clinical trial comparing intravitreal aflibercept monotherapy vs aflibercept combined with reduced fluence PDT for the treatment of polypoidal choroidal vasculopathy

PROTOCOL NUMBER:
R1735/58/2020

PROTOCOL VERSION: 8
PROTOCOL DATE: 11 November 2022

PRINCIPAL INVESTIGATOR:
Prof Gemmy Cheung Chui Ming
Senior Consultant,
Singapore Eye Research Institute
Singapore National Eye Centre
11 Third Hospital Avenue, Singapore 168751

SITE PRINCIPAL INVESTIGATOR:
A/Prof Tan Siang Hui, Colin, Senior Consultant, Tan Tock Seng Hospital
A/Prof Chee Ka Lin, Caroline, Senior Consultant, National University Hospital

CO-INVESTIGATORS:
A/Prof Ian Yeo Yew San, Senior Consultant, Singapore National Eye Centre
A/Prof Doric Wong Wen Kuan, Senior Consultant, Singapore National Eye Centre
A/Prof Lee Shu Yen, Senior Consultant, Singapore National Eye Centre
A/Prof Anna Tan Cheng Sim, Consultant, Singapore National Eye Centre
A/Prof Gavin Tan Siew Wei, Senior Consultant, Singapore National Eye Centre
Dr Ranjana Mathur, Senior Consultant, Singapore National Eye Centre
Dr Chan Choi Mun, Consultant, Singapore National Eye Centre
Dr Kelvin Teo Yi Chong, Consultant, Singapore National Eye Centre
Dr Andrew Tsai Shih Hsiang, Consultant, Singapore National Eye Centre
Dr Daniel Ting Shu Wei, Consultant, Singapore National Eye Centre
Dr Shaun Sebastian Sim Khung Peng, Consultant, Singapore National Eye Centre
Dr Fenner Beau James, Associate Consultant, Singapore National Eye Centre
Dr Chan Hiok Hong, Associate Consult ant, Singapore National Eye Centre

Dr Tan Tien-En, Senior Resident, Singapore National Eye Centre
Dr Farah Nur Ilyana Mohd Ibrahim, Senior Resident, Singapore National Eye Centre
Dr Rasipuram Chandrasekaran Priya, Clinical Fellow, Singapore National Eye Centre

A/Prof Lim Tock Han, Senior Consultant, Tan Tock Seng Hospital
A/Prof Lingam Gopal, Senior Consultant, National University Hospital
Dr Paul Zhao, Consultant, National University Hospital
Dr Chan Hwei Wuen, Consultant, National University Hospital
Dr Su Xinyi, Consultant, National University Hospital
Dr Yuen Yew Sen, Consultant, National University Hospital
Dr Thomas George, Associate Consultant, National University Hospital
Dr Naing Thet, Senior Resident Physician, National University Hospital
Dr Ariadarma Erlangga, Resident Physician, National University Hospital
Dr Mayuri Bhargava, Resident Physician, National University Hospital
Dr Wendy Wong, Resident, National University Hospital

Table of Contents

1 BACKGROUND AND RATIONALE 6

1.1 GENERAL INTRODUCTION 6
1.2 RATIONALE AND JUSTIFICATION FOR THE STUDY 6
1.2.1 RATIONALE FOR THE STUDY PURPOSE 6
1.2.2 RATIONALE FOR DOSES SELECTED 8
1.2.3 RATIONALE FOR STUDY POPULATION 8
1.2.4 RATIONALE FOR STUDY DESIGN 8

2 HYPOTHESIS AND OBJECTIVES 8

2.1 HYPOTHESIS 8
2.2 PRIMARY OBJECTIVES 9
2.3 SECONDARY OBJECTIVES 9
2.4 POTENTIAL RISKS AND BENEFITS: 9
2.4.1 POTENTIAL RISKS 9
2.4.2 POTENTIAL BENEFITS 9

3 STUDY POPULATION 10

3.1 LIST THE NUMBER AND NATURE OF SUBJECTS TO BE ENROLLED 10
3.2 CRITERIA FOR RECRUITMENT AND RECRUITMENT PROCESS 10
3.3 INCLUSION CRITERIA 10
3.4 EXCLUSION CRITERIA 11
3.5 SUBJECT REPLACEMENT 12

4 STUDY DESIGN 12

4.1 RANDOMISATION AND BLINDING 13
4.2 CONTRACEPTION AND PREGNANCY TESTING 13
4.3 STUDY VISITS AND PROCEDURES 14
4.3.1 SCREENING VISITS AND PROCEDURES 14
4.3.2 FINAL STUDY VISIT: 19
4.3.3 POST STUDY FOLLOW UP AND PROCEDURES 19
4.4 DISCONTINUATION/WITHDRAWAL 19
4.4.1 DISCONTINUATION CRITERIA 19
4.4.2 DISCONTINUATION VISIT AND PROCEDURES 20

5 TRIAL MATERIALS 21

5.1 TRIAL PRODUCT (S) 21
5.2 STORAGE AND DRUG ACCOUNTABILITY 21

6 TREATMENT 22

6.1 RATIONALE FOR SELECTION OF DOSE 22
6.2 STUDY DRUG FORMULATIONS 22
6.3 STUDY DRUG ADMINISTRATION 22
6.4 SPECIFIC RESTRICTIONS / REQUIREMENTS 23
6.5 BLINDING 23
6.6 CONCOMITANT THERAPY 23

7 SAFETY MEASUREMENTS 23

7.1 DEFINITIONS 233
7.2 COLLECTING, RECORDING AND REPORTING OF SERIOUS ADVERSE EVENTS (SAEs) TO CIRB 24

7.3 Collecting, Recording and Reporting of Serious Adverse Events (SAEs) to the Health Science Authority (HSA) 24
7.4 Safety Monitoring Plan 24
7.5 Complaint Handling 24

8 DATA ANALYSIS 24

8.1 Data Quality Assurance 24
8.2 Data Entry and Storage 24

9 SAMPLE SIZE AND STATISTICAL METHODS 25

9.1 Determination of Sample Size 25
9.2 Statistical and Analytical Plans 25

10 DIRECT ACCESS TO SOURCE DATA/DOCUMENTS 26

11 QUALITY CONTROL AND QUALITY ASSURANCE 26

12 ETHICAL CONSIDERATIONS 26

12.1 Informed Consent 26
12.2 Confidentiality of Data and Patient Records 26

13 PUBLICATIONS 27

14 RETENTION OF TRIAL DOCUMENTS 27

15 FUNDING AND INSURANCE 27

LIST OF ATTACHMENTS 28

Restricted, Sensitive (Normal)

PROTOCOL SIGNATURE PAGE

Protocol Title: A multi-center, randomized clinical trial comparing intravitreal aflibercept monotherapy vs aflibercept combined with reduced fluence PDT for the treatment of polypoidal choroidal vasculopathy

Protocol Number: R1735/58/2020

Protocol Version/ Date: 8/ 11 November 2022

Sponsor Name: National Medical Research Council (NMRC OF-LCG)

Declaration of Investigator

I confirm that I have read the above-mentioned protocol and its attachments. I agree to conduct the described trial in compliance with all stipulations of the protocol, regulations and ICH E6 Guideline for Good Clinical Practice (GCP).

Principal Investigator Name: Prof Gemmy Cheung Chui Ming

Principal Investigator Signature: ______________________

Date: 11 November 2022

1 BACKGROUND AND RATIONALE

1.1 General Introduction

Aflibercept is a fusion protein (115 kDa) consisting of VEGF-binding portions from the extracellular domains of VEGFR-1 and 2 fused to human IgG immunoglobulin that binds VEGF-A and placental growth factor (PIGF). As a solution for Intravitreal injection, 2mg of aflibercept in 50 microlitres is indicated for the treatment of neovascular (wet) age-related macular degeneration. Aflibercept was approved to treat wet AMD by FDA. It is also currently in use in Singapore to treat wet AMD.

Photodynamic therapy (PDT) utilises the photosensitive verteporfin (visudyne) in combination with an infra-red laser to induce the regression of the CNV in nAMD. The exact mechanism of action by PDT is unknown, with animal studies reporting that PDT induces endothelial cell destruction, clot formation and vascular occlusion of the choroidal neovascular complex with minimal damage to adjacent retinal structures. PDT with Verteporfin was approved by the FDA to treat wet AMD. It is also currently in use in Singapore to treat wet AMD.

1.2 Rationale and Justification for the Study

In this study, we aim to evaluate the efficacy and safety of an individualized dosing schedule comprising aflibercept and RF-PDT in patients with polypoidal choroidal vasculopathy (PCV). Favourable anatomical and visual outcomes have been shown with the use of aflibercept monotherapy in PCV and in combination therapy of PDT and ranibizumab. The objective is to compare the visual improvement from baseline to month 12, in eyes with PCV treated with aflibercept monotherapy versus combination of aflibercept with reduced fluence photodynamic therapy (RF-PDT).

Justification for drug dosage

Aflibercept 2mg dosing is approved by the FDA for the treatment of wet AMD. A PRN dosing regimen after an initial injection administered at baseline with close monthly follow up is appropriate as patients will have the option of being treatment monthly if they have very active disease or the option of prolonging their treatment interval if their disease remains quiescent.

RF-PDT has been proposed to have better safety profile in terms of less RPE and choriocapillaris damage however this has not been assessed in PCV. We hypothesize that when used in combination with afliber[illegible]pt, which has been shown to inactivate about 40-77% of polyps, we expect a good synergistic effect on [illegible]yp closure with minimal collateral damage to surrounding tissue that full-fluence PDT might otherwise cause without sacrificing therapeutic effects (augmented by aflibercept treatment).

1.2.1 Rationale for the Study Purpose

Age related macular degeneration (AMD) is one of the leading causes of blindness worldwide. In its exudative or wet form, choroidal neovascularization (CNV) causes an exudative maculopathy resulting in sudden loss of vision with severe effects on patients' quality of life. Intravitreal injections of anti-vascular endothelial growth factor agents (anti-VEGF) agents have become the mainstay of treatment for AMD CNV and has been shown to have favourable outcomes in most AMD CNV subtypes. In the Asian population, however, a particular subtype called polypoidal choroidal vasculopathy (PCV), which affects about 50% of exudative maculopathy, has been shown to have less favourable response to anti-VEGF therapy.

The best treatment option for PCV has remained unclear. Current best evidence is from 2 recent randomized controlled trials, the EVEREST II trial which compares the efficacy of ranibizumab with or without photodynamic therapy (PDT) for treatment of PCV and the PLANET trial which compares aflibercept monotherapy against a rescue PDT when aflibercept is deemed ineffective. Both trials have reported significant improvement in visual outcomes however there remain significant unanswered questions and unmet needs regarding the use of aflibercept and PDT as the best treatment for PCV.

Rationale

1. The benefit of baseline PDT was only evaluated in combination with ranibizumab in the EVEREST II study, which demonstrated superiority of combination in visual and anatomical outcome,[3] however the potential additional benefit by combining aflibercept with PDT at baseline has not been evaluated.
2. Despite good vision gains (>10 letters) in the monotherapy arm of the PLANET study, a fixed 2-monthly regimen was considered by many clinicians as an unrealistically high treatment burden. The addition of baseline PDT may reduce this injection treatment burden as shown in the EVEREST II trial for ranibizumab. (Iida T, APVRS 2016)
3. Reduced fluence PDT (RF-PDT) as opposed to full fluence PDT used in EVEREST II trial has been proposed to have better safety profile in terms of less RPE and choriocapillaris damage, but RF-PDT also has not been fully evaluated in clinical trials in PCV.

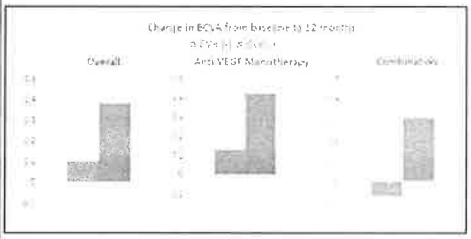

Figure 1: association of CVH and visual outcomes

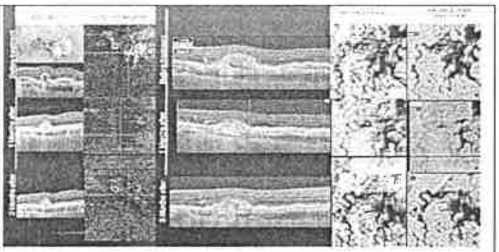

Figure 2: OCTA detected marked reduction in flow within PCV complex and deep choroidal vessels after combination therapy.

4. The EVEREST II study reported that 50.6% of patients required a low number of anti-VEGF injections in the combination arm (3-4 over 12 months). However, there are no clear baseline biomarkers can pre-identify this subgroup. A **recent post-hoc analysis for predictors of visual anatomical outcome in the EVEREST II study** showed that large polyp area at baseline had worse visual outcome with ranibizumab monotherapy compared to combination therapy (-6.9 vs +2.4 letters, p<0.001). (Cheung G, Euretina 2017). We have also reported that **choroidal vascular hyperpermeability (CVH) is associated with better visual outcome and lower injection number in combination therapy**.[4] (Fig. 1) In keeping with this observation, analysis of **choroidal vascularity index (CVI) demonstrated there is significant choroidal remodeling after treatment**. Variations in CVI characteristics may reflect different predominant pathogenic process which in turn may explain the heterogeneity in treatment responses. [5] **We have also published short-term OCTA changes after treatment. (Fig. 2)** In 90% of eyes, longitudinal changes were in agreement between OCTA and OCT. However, in 10% of eyes, OCTA was more sensitive at detecting recurrence than OCT.[6] Importantly, we have reported that reduction in flow signal in PCV complex (84.6% vs 40.0%, p=0.04) and pachyvessel calibre (75.0 vs 0.0%, p=0.01) was seen in significantly more eyes following combination vs monotherapy.[7] Increase in flow signal and/or increase in pachyvessel caliber during follow-up may be predictive of future recurrence. We will evaluate whether these OCTA response translate into lower retreatment need and better visual outcome in a 12-month longitudinal clinical trial.

In this study, we aim to compare the efficacy of combination aflibercept with RF-PDT (at baseline) and aflibercept monotherapy. This particular strategy has not been studied before and represents the amalgamation of unanswered questions from the best evidence to date for the treatment of PCV.

Hypothesis: that combination therapy of RF-PDT and aflibercept will confer better visual outcomes with reduced injection treatment burden as compared to aflibercept monotherapy.

1.2.2 Rationale for Doses Selected

The half fluence dose and regimen of verteporfin for intravenous infusion (6mg/m2) followed by laser light at a dose rate of 25J/cm2 will be used. RF-PDT has been proposed to have better safety profile in terms of less RPE and choriocapillaris damage however this has not been assessed in PCV. When used in combination with aflibercept, which has been shown to inactivate about 77% and 40% of polyps, we expect a g[illegible]d synergistic effect on polyp closure even when used with RF-PDT and reduction in collateral damage that full fluence PDT might otherwise have.

Aflibercept 2mg dosing has approved by the FDA for the treatment of wet AMD. A PRN dosing regimen after an initial injection administered at baseline with close monthly follow up is appropriate as patients will have the option of being treated monthly if they have very active disease or the option of prolonging their treatment interval if their disease remains quiescent.

1.2.3 Rationale for Study Population

We aim to recruit 160 treatment naïve patients with PCV diagnosed on ICGA. This sample population most clearly reflects the population of sight threatening PCV that would likely benefit from any insights gained from this study. This population also is similar to that chosen by prior research groups for this condition.

1.2.4 Rationale for Study Design

Multi-centred randomised controlled trial would give the best evidence in comparing the 2 treatment modalities.

2 HYPOTHESIS AND OBJECTIVES

2.1 Hypothesis

Hypothesis 1: that combination therapy of RF-PDT and aflibercept will confer better visual outcomes with reduced injection treatment burden as compared to aflibercept monotherapy.

Hypothesis 2: that combination therapy can achieve higher polyp closure rate based on indocyanine green angiography definition compared to aflibercept monotherapy

Hypothesis 3: that changes within the PCV complex and Haller's layer vessels precede exudation during follow-up.

2.2 Primary Objectives

The primary aim (1) of this study is to compare the polyp closure rate at week 12 between the 2 treatment groups.

2.3 Secondary Objectives

The secondary aims include comparing visual, anatomical, treatment burden and clinical biomarkers between each treatment group.

Aim 2: To compare the 52 weeks visual outcome between the 2 treatment groups.

Aim 3: To evaluate the developed OCTA-based retreatment indicators capable of diagnosing early subclinical recurrence.
Sub aim 3.1: To evaluate the influence of baseline choroidal vascular hyperpermeability (using novel automated quantitative software) on visual and anatomical outcome.

*For Aim 3, we will include standardized imaging protocol which incorporates SD-OCT and OCTA performed at monthly visits, as well as FA and ICGA at baseline, week 12 and week 52.

2.4 Potential Risks and Benefits:

2.4.1 Potential Risks

Combination therapy is considered standard of care and is an option when treating particular types of nAMD (such as PCV). Verteporfin PDT is another approved therapy for wet AMD. Pain, swelling, inflammation leakage into area surrounding the vein and bleeding at sight of intravenous injection. Retinal and vitreous haemorrhages have been reported as side effects of verteporfin PDT in the treatment of patients with neovascular AMD. These adverse events have been associated with temporary vision impairment. Addition of anti VEGF agents such as aflibercept may reduce the risk of haemorrhage that has been associated with verteporfin PDT via the anti-vascular permeability characteristics of aflibercept which may block vascular leakage induced by verteporfin PDT. In summary, the combination of aflibercept and verteporfin PDT may render additional therapeutic benefits to aflibercept monotherapy with mitigated risks in ocular safety. The overall risk/benefit ratio for the combination arm is expected to be more favourable than the monotherapy arm of aflibercept.

Risks to the patient would be the adverse events from the intravitreal injections like endophthalmitis, cerebral or cardiac complications, which are very rare. There is minimal risk involved in blood draw.

Patients who receive Visudyne will become photosensitive for 48 hours after the infusion. If patients have to go outdoors in daylight the first 48 hours after treatment, they must protect their skin and eyes by wearing protective clothing and dark sunglasses.

2.4.2 Potential Benefits

The benefits to the patients will be a direct benefit in receiving free intravitreal aflibercept and/or RF-PDT treatment during the course of the study depending on the treatment arm. Based on current evidence both treatment arms offer standard of care treatment.

3 STUDY POPULATION

3.1 List The Number and Nature of Subjects to be Enrolled

We will conduct a multi-centre randomized, double masked clinical trial, enrolling 160 patients with treatment naïve PCV from SNEC(n=98), TTSH(n=30) and NUH(n=32) clinics to receive either aflibercept monotherapy (n=80) or aflibercept with RF-PDT (n=80).

The study population will consist of a group of adults aged 50 and above with symptomatic macular PCV who are naïve to treatment in the study eye. Study participants can only have one study eye. If both eyes are eligible for the study, the eye without previous intravitreal anti-VEGF treatment will be selected. If both eyes are treatment naïve, the eye with worse VA should be selected as the study eye. There will be no restriction of recruitment according to the race of the patient.

3.2 Criteria for Recruitment and Recruitment Process

Potential eligibility will be assessed as part of a routine-care examination. Prior to completing any proced[illegible]s or collecting any data that are not part of usual care, written informed consent will be obtained. For potential study participants who are considered potentially eligible for the study based on a routine-care exam, the study protocol will be discussed with the potential study participant by a study investigator and study coordinator. The potential study participant will be given the informed consent form to read. Potential study participants will be encouraged to discuss the study with family members and their personal physician(s) before deciding whether to participate in the study.

3.3 Inclusion Criteria

1. Patients aged >= 50 years old at the time of informed consent.
2. Provide written informed consent.
3. Willingness and ability to comply with all scheduled visits and study procedures.
4. Confirmed diagnosis of symptomatic macular PCV based ICGA.
5. Activity of PCV confirmed by exudative activity involving the macula on OCT or FA or both.
   a) Presence of intra retinal or subretinal fluid/blood as seen on OCT
   b) Treatment naïve

*NO previous treatment with intravitreal anti-VEGF agents, regardless of the indication
*NO previous thermal laser in the macular region, or verteporfin photodynamic therapy (vPDT), regardle[illegible] of indication
*NO other previous treatment for nAMD, except oral supplements and traditional Chinese medicine

6. An ETDRS BCVA of at least 4 letters (Snellen equivalent approximately 20/800 or better) in the study eye.
7. Greatest Linear Dimension (GLD) of the total lesion area (BVN + polyps) < 5400µm (~9 MPS Disc Areas) as delineated by ICGA.

3.4 Exclusion Criteria

Participant

1. Medical condition that, in the opinion of the investigator, would preclude participation in the study (e.g. unstable medical status including blood pressure, cardiovascular disease, and glycaemic control).
2. Participation in an investigational trial within 30 days of enrolment which involves treatment with unapproved investigational drug.
3. Known allergy to any component of the study drug.
4. Blood pressure > 180/110 (systolic above 180 OR diastolic above 110 on repeated measurements). If blood pressure is brought below 180/110 by anti-hypertensive treatment, individual can become eligible.
5. Myocardial infarction, other acute cardiac event requiring hospitalization, stroke, transient ischemic attack, or treatment for acute congestive heart failure within 4 months prior to randomization.
6. Systemic anti-VEGF or pro-VEGF treatment within four months prior to randomization or anticipated use during the study.
7. Amblyopia or blind in one eye

Study Eye

1. Eye with intra retinal or subretinal fluid due to other causes than PCV
2. An ocular condition is present (other than PCV) that, in the opinion of the investigator, might affect intra or sub retinal fluid or alter visual acuity during the course of the study (e.g., DME, vein occlusion, uveitis or other ocular inflammatory disease, neovascular glaucoma, etc.)
3. Substantial cataract that, in the opinion of the investigator, is likely to be decreasing visual acuity by more than three lines (i.e., cataract would be reducing acuity to worse than 20/40 if eye was otherwise normal).
4. Any intraocular surgery within 1 month of enrolment
5. Treatment with intra-vitreal corticosteroids
6. History of retinal detachment or surgery for retinal detachment
7. History of vitrectomy
8. History of macular hole
9. Evidence of vitreomacular traction that may preclude resolution of macular oedema > 4 disc areas of intra/sub retinal haemorrhage
10. Aphakia
11. Exam evidence of external ocular infection, including conjunctivitis, chalazion, or significant blepharitis

Other Eye

1. Active intraocular inflammation
2. History of uveitis

3.5 Subject Replacement

There will be no replacement of subjects who drop out of the study.

4 STUDY DESIGN

We plan for a Multi-center randomized, double-masked clinical trial.
Expected duration: 52 weeks
Study design is summarized in figure 3 and details are as follows in this section

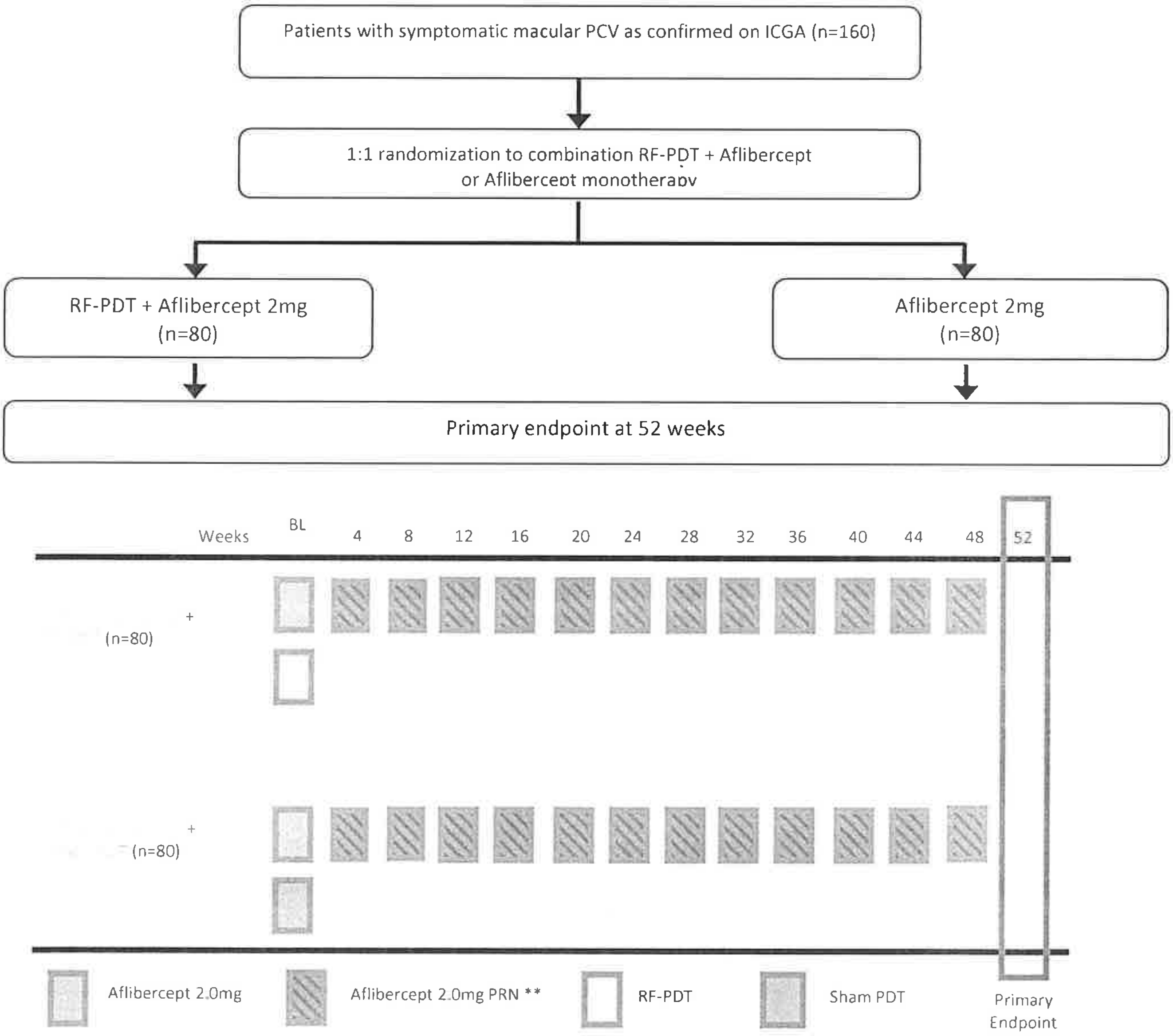

**Aflibercept administered PRN as per protocol-specific retreatment criteria if there was a BCVA loss or presence of disease activity as seen on OCT. The interval between 2 aflibercept treatments must be least 21 days.

Restricted. Sensitive (Normal)

Figure 4. Retreatment criteria after baseline treatment

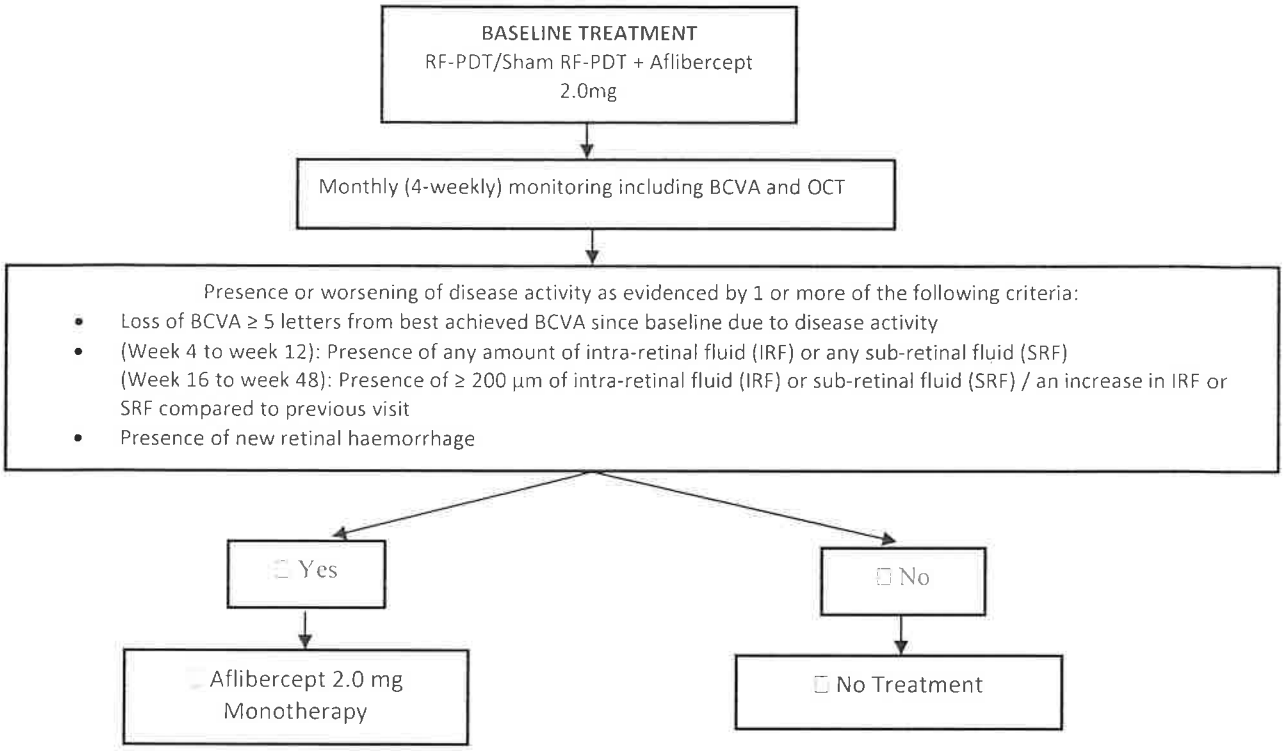

4.1 Randomisation and Blinding

Study participants will be randomized to 2 treatment groups using a ratio of 1:1. Randomization will be performed using a blocked randomization method. Each site will be randomized to a 1:1 ratio for each study treatment arm.

SNEC site will be randomized in blocks of 20 and the remaining 18 participants will be randomized in blocks of 9. For TTSH site, 30 participants will be randomized in blocks of 10. For NUH site, 32 participants will be randomized in block of 20, with the remaining 12 participants randomized in blocks of 6.

SNEC site will be providing randomization for NUH and TTSH sites. Both participants and masked team will be masked to treatment received.

4.2 Contraception and Pregnancy Testing

N/A

4.3 Study Visits and Procedures

Subjects will be closely monitored by the investigators. Tests procedures including slit lamp examination, dilated fundus examination and intraocular pressure (IOP) checks, which are part of standard of care, will be performed on every study visit.

At each visit, subjects will be assessed based on best corrected visual acuity (BCVA), ophthalmic examination, OCT and OCT-angiograph (OCT-A). In addition, colour photography, autofluorescence photography, fluorescein and indocyanine green angiography will be performed at baseline, week 12 and week 52. Additional investigations at interim visits may be performed at the discretion of the investigator if there is evidence of disease activity.

• Qualification for retreatment with Aflibercept (fig 4) (week 4 to week 48) will be based on signs of disease activity defined as persistent intraretinal or subretinal fluid on OCT and BCVA.

• At week 52, all subjects will return for end of study visit. All subjects will be assessed based on BCVA, ophthalmic examination, FA, ICGA, colour fundus photography, Autofluorescence and OCT and OCT[illegible]

4.3.1 Screening Visits and Procedures

Screening Evaluation and Baseline Testing

Historical Information
A history will be elicited from the potential study participant and extracted from available medical records. Data to be collected will include: age, gender, ethnicity and race, past medical history and medications being used, as well as ocular diseases, surgeries, and treatment.

An assessment of visual related quality of life will be performed using the impact of visual impairment (IVI) questionnaire.

Baseline Testing Procedures
The following procedures are needed to assess eligibility and/or to serve as baseline measures for the study.
If a procedure has been performed (using the study technique and by delegated personnel) as part of usual care, it does not need to be repeated specifically for the study if it was performed within the defined time (within 21 days prior to baseline) windows specified below.

1. Best-corrected Visual Acuity: BCVA will be measured using the ETDRS VA protocol following manifest refraction.
2. Blood Sample (optional): 27mls of blood serum and DNA sample will be obtained for assessment of biomarkers and genetics. [SNEC site only]
3. Optical Coherence Tomography/ OCT Angiography: OCT and OCTA will be performed. Both standard and enhanced depth imaging scans will be performed.
4. Ocular examination on each eye including slit lamp, measurement of intraocular pressure, lens assessment, and dilated fundus examination (within 21 days prior to randomization).
5. Blood pressure measurements
6. Fundus Photography, Autofluorescence photography
7. Fundus fluorescein and Indocyanine Green angiography: FFA and ICGA will be performed.
8. Home Monitoring Mobile app assessment (Alleye) (optional) [SNEC site only]

Disease characteristics of the study eye assessed by the investigator at screening (day 1):

- Diagnosis of PCV based on ICGA.
- Presence of activity clinically as evidence by presence of haemorrhage, edema.
- Presence of activity as evidence by intra retinal or sub retinal fluid on OCT.

Study visits & Procedures

Procedure/ Assessments	Screening	Baseline	WK 4	WK 8	WK 12	WK 16	WK 20	WK 24	WK 28	WK 32	WK 36	WK 40	WK 44	WK 48	WK 52 Last visit
Visit	1	2	3	4	5	6	7	8	9	10	11	12	13	14	15
Days	-14 to 1	1	28	56	84	112	140	168	196	224	252	280	308	336	364
Visit window (Days)			±7	±7	±7	±7	±7	±7	±7	±7	±7	±7	±7	±7	±7
Clinic Consultation (including slit lamp and dilated fundus examinations)	X	X	X	X	X	X	X	X	X	X	X	X	X	X	X
Informed Consent	X														
IVI		X													X
Vital Signs	X	X			X										X
FFA	X				X										X
ICGA	X				X										X
BCVA	X	X	X	X	X	X	X	X	X	X	X	X	X	X	X
IOP	X	X	X	X	X	X	X	X	X	X	X	X	X	X	X
OCT	X	X	X	X	X	X	X	X	X	X	X	X	X	X	X
OCTA	X	X	X	X	X	X	X	X	X	X	X	X	X	X	X
Color fundus Photography	X				X										X
Auto fluorescence	X				X										X
Blood sample(optional) [SNEC Site only]		X													X
Home Monitoring Mobile app (Alleye) (optional) [SNEC site only]	X	X	X	X	X	X	X	X	X	X	X	X	X	X	X
Transport allowance	X	X	X	X	X	X	X	X	X	X	X	X	X	X	X
IVT treatment (Eylea)		X	Per retreatment criteria (Figure 4)												
Verteporfin/sham RF-PDT (80 pts/ 80 pts)		X													

Screening (visit 1) and baseline (visit 2) can be done on the same day at PI/ Co-I discretion.

Home Monitoring Mobile App (Alleye) [optional]

This smartphone based app (Alleye) is a CE (European Conformity) – marked Class I device that received FDA (Food and Drug Administration) 510(k) clearance for monitoring eyesight in age related macular degeneration(AMD) in 2018. It was approved for use in patients with AMD, for both detection of new disease

and monitoring of treatment response.

Participants with smart phone will be encouraged to download and utilize this mobile medical software application for the detection and characterisation of metamorphosia in macular diseases which can occur in AMD, epiretinal membrane, diabetic retinopathy and macular edema, and any other cause of macular edema including retinal vein occlusion etc.

The alleye app was designed for self-directed use in the home, by patients who can regularly perform the test on their personal mobile devices. Patients use monocular vision to complete the digital task, which is to align a centre dot to 2 fixed flanking dots to create an imaginary straight line. (Figure 1) This task is repeated 12 times with flanking dots in different orientations and distances. The app displays a score reflecting the visual performance of the patient in relation to a healthy individual. The maximum score is 100 and minimum is 0. A score of 100 indicates visual performance comparable to a healthy individual with no eye conditions. We will monitor changes in score over time for subjects who perform the app. The Alleye app was designed for self-directed use in the home, by patients who can regularly perform the test on their personal mobile devices. The Alleye app does not constitute diagnosis or treatment of AMD and also cannot replace them.

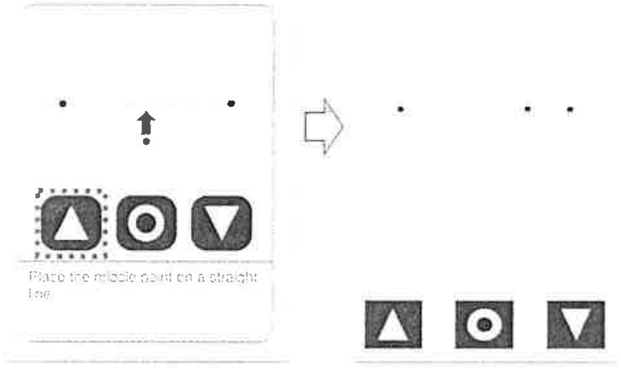

(Figure 1)

Treatment Procedures

Aflibercept (Eylea) Intravitreal Injections
Study eyes will receive a dose of 2mg in 0.05 ml of aflibercept.

Intravitreal Injection Technique
Antibiotics in the pre-, peri-, or post-injection period are not necessary but can be used at investigator discretion if such use is part of his/her usual routine.

Prior to the injection, the study eye will be anaesthetized with topical anaesthetic, followed by a povidone iodine prep of the conjunctiva. (Instil 5% povidone iodine on to the ocular surface and allow adequate time prior to injection)

Aflibercept will be withdrawn using aseptic technique through an 18-guage filter needle attached to a 1-ml syringe. The needle will be discarded after withdrawal of the vial contents and should not be used for intravitreal injection. The needle should be replaced by a sterile 30-guage needle for the intravitreal injections. The contents of the syringe should be expelled until the plunger is aligned with the line that marks 0.05ml on the syringe.

The injection will be performed using sterile technique. Investigator will use a surgical hand disinfection

technique and wear sterile gloves. Periocular skin and eyelid margins and eye lashes will be cleaned with 5-10% povidone iodine.

Skin will be dried and drape will be applied. Investigator will insert eyelid speculum, ensuring that it is well positioned underneath the eyelids to direct the eyelashes away from the field. Callipers should be used to mark the injection site. The entry site of the needle should be 3.0-3.5 mm from the limbus in pseudophakic patients, and 3.5-4.0 mm in phakic patients.

The conjunctiva may be displaced anteriorly using either forceps or cotton tipped applicator so that no direct route between vitreous and ocular surface remains. The needle is inserted perpendicular through sclera with the tip aimed towards the centre of the globe (to avoid any contact with the posterior lens).

IOP measurement post-injection is not mandatory. While small volume injections (0.05ml) are unlikely to cause IOP rise, it should be considered in patients with ocular hypertension or glaucoma, and in all cases where patients are symptomatic for pain or reduced vision immediately following injection. Should a high intraocular pressure resulting in non-perfusion of the central retinal artery occur, indicated by no perception of light (NPL) in the treated eye, an anterior chamber paracentesis is indicated. Such decompression needs to be achieved within 3-5 minutes. Patients should be instructed to report any symptoms regarding eye pain or discomfort, increased redness of the eye, or additional blurring of vision (which may indicate endophthalmitis) to the treating ophthalmologist without delay.

Intravitreal injections will be performed by the delegated Investigators (includes Consultants and Residents enrolled as investigators in the study).

Delay in Giving Injections

If a scheduled injection is not given on the day of study visit, it may be administered within 7 days after the occurrence of the study visit. If it is not given by that time, it will be considered missed. If an injection is given late, the next scheduled injection should occur no sooner than 21 days after the previous injection.

Non-Study Eye Injections

If the non-study eye is going to be treated for any condition which requires treatment with an anti-VEGF agent, it may be treated at the discretion of the investigator according to the standard of care and the study will not bear the cost for treatment of the non-study eye. Treatment of the fellow-eye with aflibercept is possible.

Treatment Regimen Adjustments

If the study eye develops a treatment-related adverse event at any time during the study, treatment dose may be temporarily held and the reason for dose holding will be recorded in the CRF.

The treatment regimen will be adjusted based on the following criteria:

• Intraocular inflammation: may hold dose at the investigator's discretion, eg, if intraocular inflammation is >= 2+ in the study eye. Treatment may resume when the inflammation has resolved.

• IOP: hold dose if IOP is >= 30 mm Hg in the study eye. Treatment may resume when IOP is <= 30 mm Hg, either spontaneously or by treatment, as determined by evaluating physician.

• New retinal break or retinal detachment: hold dose for the study eye. Treatment may resume after the retinal

break/detachment had been successfully treated.

• Ocular and/or periocular infection: hold dose until the infection is resolved in both eyes.

The investigator may hold or discontinue study treatment for other safety reasons at his/her discretion.

Warnings and Precautions

Injection Procedure-related Reactions

Treatments such as aflibercept that are administered via intravitreal injections can be associated with a number of adverse events. These include conjunctival haemorrhage, conjunctival hyperaemia, eye irritation, eye pain, eye pruritus, endophthalmitis, foreign body sensation in eyes, intraocular inflammation, rhegmatogenous retinal detachment, retinal tear, increases in IOP, vitreous detachment, and iatrogenic traumatic cataract.

Intraocular Inflammation, Endophthalmitis, and Retinal Detachments

Intravitreal injections have the potential to be associated with intraocular inflammation, infectious and non-infectious endophthalmitis, and retinal detachments which could be sight-threatening. Proper aseptic injection technique should always be used when administering aflibercept. In addition, patients should be monitored following the injection to permit early treatment should inflammation, infection, or retinal detachment occur. Patients should be informed that in the days following administration of aflibercept intravitreally, patients are at risk for the development of endophthalmitis. If the eye becomes red, sensitive to light, painful, or develops a worsening in vision, the patient should seek immediate care with their ophthalmologist.

Increases in Intraocular Pressure

Increases in IOP have been noted following intravitreal injection with anti-VEGF agents. The increase in IOP is typically transient in nature and is probably due to the volume increase in the eye after injection. Therefore, IOP as well as the perfusion of the optic nerve head should be monitored and managed appropriately following the intravitreal injection.

Cataract Formation
Intravitreal injections have the potential to be associated with iatrogenic traumatic cataract formation.

Anti-VEGF Agents Class Effects

Hypertension, non-ocular haemorrhage and thromboembolic events have been reported with systemic anti-VEGF therapy. There is a potential risk of arterial thromboembolic events following intravitreal use of inhibitors of VEGF. Arterial thromboembolic events are defined as nonfatal stroke, nonfatal myocardial infarction, or vascular death (including deaths of unknown causes).

Verteporfin RF-PDT/ Sham RF-PDT administration

Procedure room preparation

Treatment will commence on Day 1 of the study with both verteporfin RF-PDT/ sham RF-PDT and Aflibercept. The procedure room for verteporfin (Visudyne) / sham RF-PDT will be prepared first.

Pre-treatment patient preparation

The verteporfin (Visudyne) / sham (5% dextrose in water solution for infusion) intravenous infusion must be administered using standard aseptic technique. The skin at the infusion site must be disinfected prior to the infusion as per local standard.

This intravenous procedure will be done using the same cannula inserted into patient for FFA/ICG procedure. There will be no additional insertion of cannula. During the infusion, the delivery syringe and intravenous line should be wrapped in aluminium to mask the identity of treatment. The active RF-PDT/ Sham RF-PDT will be performed by an unmasked investigator enrolled of the study team.

Active RF-PDT

A dosage of Verteporfin calculated at 6mg/m^2 body surface area in a 30-ml solution will be infused intravenously over a 10-minute period. Fifteen minutes after the start of the infusion, the study eye will be anaesthetized with topical anaesthetic, and a contact lens will be used for the laser procedure. Laser light is applied to the study eye for 83 seconds with the parameters:

- Light dose (reduced fluence) 25 J/cm^2
- Light wavelength 689 nm

Sham RF-PDT

This sham procedure is the same as that used in the EVEREST II RCT (2013-2015) - [CIRB No: 2013-271-A] and PLANET RCT (2014-2015) - [CIRB: 2014/245/A]. These are the landmark PCV trials performed in year 2013 – 2015

The sham infusion is prepared using 5% of dextrose in water solution. A 30ml of the sham solution will be infused intravenously over a period of 10 minutes to mimic verteporfin infusion. After the infusion, topical anaesthetic eye drop will be used to numb the study eye and a special contact lens will then be placed over the eye for laser procedure. Following the eye drop application, sham laser procedure that mimics the active RF-PDT procedure will be performed. Laser aiming beam applied with settings at 0 fluence covering the size of the lesion will be used to maintain the masking of the sham procedure. There will be no therapeutic / damaging effect on retina tissue as no verteporfin or laser power is administered.

Post treatment care

Patients who receive verteporfin RF-PDT will become temporarily photosensitive after the infusion. All patients who receive verteporfin RF-PDT/ sham RF-PDT should be instructed to avoid direct sunlight for 48 hours.

Possible side effects for Active RF-PDT

Some patients may experience adverse reactions. These reactions may include:

- Pain, swelling, inflammation, leakage into the area surrounding the vein and bleeding at the intravenous injection site

- Corneal abrasion related to the use of contact lens in the procedure.
- Blurred vision and other visual disturbances.
- Transient back pain during infusion
- Severe vision loss, though partial recovery occurs in some patients
- Photosensitivity during the first two days after therapy

Possible side effects for Sham RF-PDT

- Pain, swelling, inflammation, leakage into the area surrounding the vein and bleeding at the intravenous injection site.
- Corneal abrasion related to the use of contact lens in the procedure.
- Since a true laser light and Verteporfin (Visudyne) is not used during the Sham RF-PDT, there will be no side effects related to the chemical reaction of laser and (Visudyne).

Optional Blood collection (Baseline & Week 52) [SNEC site only]

Blood test (27 mLs) will be collected from participants at baseline visit and week 52 visit (optional). The st[illegible]ed samples will be aimed for the purpose of storing serum (for biomarker studies) and DNA (for genetic ma[illegible]er studies). Confidentiality will be protected by assigning non-identifier labelling. In such cases the results may not provide any direct health benefits to the participants. Unused samples will be stored for 15 years for future research as permitted by the IRB, with consent from the participants.

4.3.2 Final Study Visit:

Last visit will occur at week 52 (+/-7 days)
No special procedures will be carried out in addition to that stated in the schedule.

4.3.3 Post Study Follow up and Procedures

Study participants will continue their routine follow-up with their regular physicians upon completion of the final study visit. There will be no post study visits planned.

4.4 Discontinuation/Withdrawal

4.4.1 Discontinuation Criteria

The study may be discontinued by the Data and Safety Monitoring Committee [DSMC] prior to the preplanned completion of follow-up for all study participants.

4.4.2 Discontinuation Visit and Procedures

Participants may voluntarily withdraw from the study at any time. If a study participant is considering withdrawal from the study, the principal investigator should personally speak to the individual about the reasons, and every effort should be made to accommodate him or her.

Study participants who withdraw will be asked to have a final closeout visit at which the testing described for the protocol visits will be performed. Study participants who have an adverse effect attributable to a study treatment or procedure will be asked to continue in follow-up until the adverse event has resolved or stabilized.

5 TRIAL MATERIALS

5.1 Trial Product (s)

Aflibercept (EYLEA) is solution for injection, clear, colourless to pale yellow, iso-osmotic solution, pH6.2.

Each Visudyne vial contains 15mg of verteporfin. After constitution, 1 mL contains 2mg of verteporfin. 7. 5mL of reconstituted solution contains 15mg of verteporfin.

Information on trial product is detailed in the package insert as attached in Appendix.

Sham RF-PDT consists of dextrose 5% solution followed by light application RF-PDT.

The sham infusion will be prepared with 5% dextrose in water solution for infusion. A 30 mL volume of this solution is infused intravenously over a 10 minute period to mimic the verteporfin infusion.

5.2 Storage and Drug Accountability

Site: Singapore Eye Research Institute:
Aflibercept (EYLEA) will be stored in a refrigerator at 2°C to 8°C. It will be stored securely in the SERI Pharmacy, SNEC building. It will be monitored by a 24-hour Temperature monitoring System (TMS) and report will be generated on a bio-weekly basis. Alarms to any excursion will be triggered and the designated staff will be informed.

Visudyne will be stored in the original package in order to protect from light, at a room temperature not above 25°C. It will be stored securely in the SERI Pharmacy cabinet with restricted access.

Site: Tan Tock Seng Hospital:
Aflibercept (EYLEA) will be stored in a refrigerator at 2°C to 8°C. It will be stored securely with temperature monitoring. Alarms to any excursion will be triggered and the designated staff will be informed.
Visudyne will be stored in the original package in order to protect from light at temperature not above 25°C. It will be stored securely with temperature monitoring. Alarms to any excursion will be triggered and the designated staff will be informed.

Site: National University Hospital:

Aflibercept (EYLEA) will be stored in a refrigerator at 2°C to 8°C. It will be stored securely with temperature monitoring. Alarms to any excursion will be triggered and the designated staff will be informed.

Visudyne will be stored in the original package in order to protect from light at temperature not above 25°C. It will be stored securely with temperature monitoring. Alarms to any excursion will be triggered and the designated staff will be informed.

The label on each Aflibercept/ Visudyne/ Dextrose 5% will include short study title, the name and country of

origin of the manufacturer, batch number, trial number, expiry date, storage conditions, emergency contacts, subject no/ initials, date of dispensed, visit/ week and the words "For Clinical Trial Use Only".

6 TREATMENT

6.1 Rationale for Selection of Dose

RF-PDT has been proposed to have better safety profile in terms of less RPE and choriocapillaris damage however this has not been assessed in PCV. When used in combination with aflibercept, which has been shown to inactivate about 40-77% of polyps, we expect a good synergistic effect on polyp closure even when used with RF-PDT and reduction in collateral damage that full fluence PDT might otherwise have.

Aflibercept 2mg dosing has approved by the FDA for the treatment of wet AMD. A PRN dosing regimen after an initial injection at baseline with close monthly follow up is appropriate as patients will have the option of being treatment monthly if they have very active disease or the option of prolonging their treatment interval if their disease remains quiescent.

6.2 Study Drug Formulations

RF-PDT will be administered on Day 1 visit as per randomization group. Verteporfin, the sensitising agent will be administering intravenously followed by the activation laser.

Aflibercept will be administered as an intravitreal injection. Procedures are also outlined in section 1.2.2

6.3 Study Drug Administration

Aflibercept intravitreal injection will be administered as per label use for nAMD. A 2mg dosing has approved by the FDA for the treatment of wet AMD. A PRN dosing regimen of an initial injection administered at baseline with close monthly follow up will be administered. Patient's treatment will vary according to their disease activity. This tailored treatment regimen will result in more monitoring visits but treatments only when necessary. There are no special precautions or warnings specific to the administration of aflibercept in this trial.

RF-PDT has been proposed to have better safety profile than full fluence PDT in terms of less RPE and choriocapillaris damage however this has not been assessed in PCV. When used in combination with aflibercept, which has been shown to inactivate about 40-77% of polyps, we expect a good synergistic effect on polyp closure with reduced collateral damage.

Patients will be monitored at monthly visits over a period of one year and treated with Aflibercept and verteporfin RF-PDT/sham RF-PDT on Day 1. Aflibercept is to be administered after the verteporfin RF-PDT/sham RF-PDT treatment. All reasonable attempts must be made to treat a patient meeting all retreatment criteria. If the treatment is not administered, the patients must still be scheduled for the next visit according to the protocol.

<u>Aflibercept (EYLEA)</u>

Aflibercept will be administered monthly as an intravitreal injection with a standard dose of 2mg in 0.05 ml. The interval between aflibercept treatments will be adjusted as appropriate according to the disease activity. The interval between two aflibercept doses should not be shorter than 21 days.

In the maintenance phase, clinical examination including visual acuity should be performed on a monthly basis and the necessity of administration of aflibercept should be evaluated based on the results of examination and patient condition (refer to figure 4 retreatment criteria). Efficacy should be regularly assessed and aflibercept therapy should not be continued aimlessly if efficacy is no longer observed.

Verteporfin RF-PDT/ Sham RF-PDT

On day 1, verteporfin RF-PDT/ sham RF-PDT is to be administered. Only one laser spot will be delivered to the macular. Additional information on how to laser can be found the verteporfin label.

Any verteporfin RF-PDT/ sham RF-PDT treatment must be administered within 14 days of the FFA/ICGA.

6.4 Specific Restrictions / Requirements

None

6.5 Blinding

Since the two study drugs have very different appearances and route of administration, study treatment masking during the study is necessary to minimise the potential for patient and investigator bias.

Blinding will be carried out for the administration of RF-PDT. Study participants will undergo RF-PDT and sham RF-PDT depending on treatment arm randomisation result. All RF-PDT procedures will be carried out in an identical fashion with preparation of drug performed by unmasked coordinator/investigator. During the procedure, the drug administration set will be covered to ensure that patient is not aware of treatment administered.

The treating physician, unmasked study coordinators will help with the preparation and administration of the treatment will be masked.

In case of emergency that requires code-break, unmasked coordinator(s) and investigator(s) will refer to the randomisation list in unmasked Investigator file. An assessment will be done by the unmasked Investigators after an emergency unmasking to assess if study drug should be discontinued for a given patient.

6.6 Concomitant therapy

All medications (prescription and over the counter), vitamin and mineral supplements, and / or herbs taken by the participant will be documented.

7 SAFETY MEASUREMENTS

7.1 Definitions

An adverse event (AE) is any untoward medical occurrence in a patient or clinical investigation subject administered a pharmaceutical product and which does not necessarily have a causal relationship with this treatment. An adverse event (AE) can therefore be any unfavourable and unintended sign (including an abnormal laboratory finding), symptom, or disease temporally associated with the use of a medicinal (investigational) product, whether or not related to the medicinal (investigational) product.

A serious adverse event (SAE) is any untoward medical occurrence that at any dose:

- results in death
- is life-threatening
- requires inpatient hospitalisation or prolongation of existing hospitalisation
- results in persistent or significant disability/incapacity, or
- is a congenital anomaly/birth defect

7.2 Collecting, Recording and Reporting of Serious Adverse Events (SAEs) to CIRB

Only related SAEs (definitely/ probably/ possibly) will be reported to CIRB. Related means there is a reasonable possibility that the event may have been caused by participation in the clinical trial. Please refer to the CIRB website for more information on Reporting Requirement and Timeline for Serious Adverse Events.

The investigator is responsible for informing CIRB after first knowledge that the case qualifies for reporting. Follow-up information will be actively sought and submitted as it becomes available.

Related AEs will not be reported to CIRB. However, the investigator is responsible to keep record of such AEs cases at the Study Site File.

7.3 Collecting, Recording and Reporting of Serious Adverse Events (SAEs) to the Health Science Authority (HSA)

All SAEs that are unexpected and related to the study drug will be reported to HSA. Please refer to the HSA website for more information on Safety Reporting Requirements for Clinical Trials.

7.4 Safety Monitoring Plan

A Data and Safety Monitoring Committee will approve the protocol, template informed consent form, and substantive amendments and provide independent monitoring of adverse events. Cumulative adverse event data are semi-annually tabulated for review by the DSMC. Following each DSMC data review, a summary will be made available for submission to Institutional Review Board. A list of specific adverse events to be reported to the DSMC expeditiously will be compiled and included as part of the DSMC Standard Operating Procedures.

7.5 Complaint Handling

Complaints will be handled by the PI and study coordinators and if required the Quality Assurance team at the Singapore National Eye Centre.

8 DATA ANALYSIS

8.1 Data Quality Assurance

The Investigator(s)/ Singapore National Eye Centre will permit study-related monitoring audits, MCRC and or EC review and regulatory inspection(s), providing direct access to source data/ document.

8.2 Data Entry and Storage

Research data will be filled in case report forms and entered onto a specifically designed database using Redcap. Data will be encrypted with password and kept in controlled shared folder where confidentiality and

participant privacy will be maintained. The data will be coded and the participants will be de-identified and the master list will be encrypted with the password. Case files will be under lock and key, with restricted access to the key, as defined within the study delegation log.

9 SAMPLE SIZE AND STATISTICAL METHODS

9.1 Determination of Sample Size

We have sought statistical advice and based on a previous study of polyp closure in PCV patients, we estimate the closure rate to be 40% in the aflibercept arm. To detect superiority of half fluence PDT + aflibercept arm (assuming closure rate of 75%), a sample of 25 patients in each arm will have 80% power to detect the difference with a 10% confidence level. Furthermore, this same sample size will detect a difference in number of injections of 2 based on reducing from 7.3 in the monotherapy arm vs 5.2 in the combination arm with >99% power (injection numbers are based on EVEREST II study data).

9.2 Statistical and Analytical Plans

Statistical Methods and Sample Size Calculation

For statistical purposes, baseline will be defined as the last available non-missing value collected just prior to the start of treatment in the study eye. For patients with screening assessments but who do not enter the treatment period, data will only be listed. For all patients only one eye will be considered as the study eye, and only for this eye efficacy analysis will be performed. Unless otherwise specified, all statistical tests will be two-sided with a 0.05 level of significance, and all confidence intervals will be two-sided with 95% confidence level.

Categorical variables will be presented as the number and percentage of patients in each category. Continuous variables will be summarized using descriptive statistics (e.g. n, mean, standard deviation, median, minimum, and maximum). Descriptive statistics will be provided for patient demographics and all baseline characteristics. Relevant medical history and current medical conditions will be tabulated by system organ class and preferred term of the MedDRA dictionary. Separate tables will be provided for ocular and non-ocular histories and conditions. Full analysis set (FAS) comprises all patients to whom treatment regimen has been assigned.

Intent-to-treat (ITT): patients will be analyzed according to the treatment regimen they are assigned to at randomization. No data will be excluded from the FAS analyses because of protocol deviation. All efficacy evaluations will be carried out on the FAS.

Per protocol Set (PPS) will consist of all patients in the FAS who followed the treatment regimen as randomized and completed Week 24 without clinically significant protocol deviations.

Primary efficacy evaluation will be carried out on both the FAS and the PP set. The primary variable is the proportion of eyes with polyps closure at 3 months. The statistical testing will be carried out using Chi-square test. The analysis will be repeated for the PP set using the same model.

Analysis of secondary endpoints will focus on the study eye only and will be based on the FAS. At all the time points assessed, each efficacy variable will be presented graphically and descriptive statistics provided based on absolute values and changes from baseline. For continuous and ordered categorical variables, changes from baseline will be compared between treatment groups using ANOVA.ANCOVA models/ t-test and

stratified/unstratified Cochran-mantel-Hansel tests. Stratification will follow the approach described for the primary analysis as applicable. Logistic regression will be used for analyses of binary endpoints.

10 DIRECT ACCESS TO SOURCE DATA/DOCUMENTS

The investigator(s)/institution(s) will permit study-related monitoring, audits and/or IRB review and regulatory inspection(s), providing direct access to source data/document.

11 QUALITY CONTROL AND QUALITY ASSURANCE

The Investigator(s)/ Singapore National Eye Centre will permit study-related monitoring audits, MCRC and or EC review and regulatory inspection(s), providing direct access to source data/ document.

12 ETHICAL CONSIDERATIONS

This study will be conducted in accordance with the ethical principles that have their origin in the Declaration of Helsinki and that are consistent with the Good Clinical Practice and the applicable regulatory requirements.

This final Clinical Trial Protocol, including the final version of the Participant Information Sheet and Consent Form, must be approved in writing by the Centralised Institutional Review Board (CIRB) and regulatory approval from Health Sciences Authority (HSA), prior to enrolment of any patient into the study.

The principle investigator is responsible for informing the CIRB and HSA of any amendments to the protocol or other study-related documents, as per local requirement.

12.1 Informed Consent

Potential eligibility will be assessed as part of a routine-care examination. Prior to completing any procedures or collecting any data that are not part of usual care, written informed consent will be obtained. For potential study participants who are considered potentially eligible for the study based on a routine-care exam, the study protocol will be discussed with the potential study participant by a study investigator.

The potential study participant will be given the Informed Consent Form to read. Potential study participants will be encouraged to discuss the study with family members and their personal physician(s) before deciding whether to participate in the study. In obtaining and documenting informed consent, the investigator will comply with the GCP guidelines and to the ethical principles that have their origin in the Declaration of Helsinki.

12.2 Confidentiality of Data and Patient Records

Research data will be anonymized as soon as possible and both identification key and the de-identified data will store in separate folders in the SERI access-controlled shared folders. Case files will be kept under lock and key, with restricted access to the key, as defined within the study delegation log.

13 PUBLICATIONS

The publications will report findings relating to the aims of the research study. All investigators will be given the opportunity to contribute as authors on various publications. Name order will be based on their respective contributions. No publication will be submitted without consent from all co-authors.

14 RETENTION OF TRIAL DOCUMENTS

All clinical research documentation/ records will be retained for 15 years post study completion.

15 FUNDING and INSURANCE

This study is supported by NMRC (OF-LCG)/ SERI and covered under the National Clinical Trial (CT) Insurance policy.

Restricted. Sensitive (Normal)

List of Attachments

1. Miller JW, Walsh AW, Kramer M, et al. Photodynamic therapy of experimental choroidal neovascularization using lipoprotein-delivered benzoporphyrin. *Arch Ophthalmol.* 1995;113(6):810-818.
2. Kramer M, Miller JW, Michaud N, et al. Liposomal benzoporphyrin derivative verteporfin photodynamic therapy. Selective treatment of choroidal neovascularization in monkeys. *Ophthalmology.* 1996;103(3):427-438.
3. Koh A, Lai TYY, Takahashi K, et al. Efficacy and Safety of Ranibizumab With or Without Verteporfin Photodynamic Therapy for Polypoidal Choroidal Vasculopathy: A Randomized Clinical Trial. *JAMA Ophthalmol.* 2017.
4. Yanagi Y, Mohla A, Lee WK, et al. Prevalence and Risk Factors for Nonexudative Neovascularization in Fellow Eyes of Patients With Unilateral Age-Related Macular Degeneration and Polypoidal Choroidal Vasculopathy. *Invest Ophthalmol Vis Sci.* 2017;58(9):3488-3495.
5. Ting DSW, Yanagi Y, Agrawal R, et al. Choroidal Remodeling in Age-related Macular Degeneration and Polypoidal Choroidal Vasculopathy: A 12-month Prospective Study. *Sci Rep.* 2017;7(1):7868.
6. Wei X, Ting DSW, Ng WY, Khandelwal N, Agrawal R, Cheung CMG. CHOROIDAL VASCULARITY INDEX: A Novel Optical Coherence Tomography Based Parameter in Patients With Exudative Age-Related Macular Degeneration. *Retina.* 2017;37(6):1120-1125.
7. Teo KYC, Yanagi Y, Lee SY, et al. Comparison of Optical Coherence Tomography Angiographic Changes after Anti-Vascular Endothelial Growth Factor Therapy Alone or in Combination with Photodynamic Therapy in Polypoidal Choroidal Vasculopathy. *Retina.* 2017.
8. Hirami Y, Tsujikawa A, Otani A, et al (2007) Hemorrhagic complications after photodynamic therapy for polypoidal choroidal vasculopathy. Retina; 27:335-41.
9. Ojima Y, Tsujikawa A, Otani A, et al (2006) Recurrent bleeding after photodynamic therapy in polypoidal choroidal vasculopathy. Am J Ophthalmol; 141:958-60.
10. Prakash M, Han DP (2006) Recurrent bullous retinal detachment from photodynamic therapy for polypoidal choroidal vasculopathy. Am J Ophthalmol; 142:1079-81.
11. Yodoi Y, Tsujikawa A, Otani A, et al (2007) Chorioretinal anastomosis after photodynamic therapy for polypoidal choroidal vasculopathy: CRA after PDT for PCV. Int Ophthalmol; 28:297-9.
12. COMBINED PHOTODYNAMIC THERAPY WITH VERTEPORFIN AND INTRAVITREAL BEVACIZUMAB FOR CHOROIDAL NEOVASCULARIZATION IN AGE-RELATED MACULAR DEGENERATION DHALLA, MANDEEP S. MD[*†]; SHAH, GAURAV K. MD[*†]; BLINDER, KEVIN J. MD[*†]; RYAN, EDWIN H. JR MD[‡]; MITTRA, ROBERT A. MD[‡]; TEWARI, ASHEESH MD[*†]
13. Verteporfin Therapy and Intravitreal Bevacizumab Combined and Alone in Choroidal Neovascularization due to Age-Related Macular Degeneration RatimirLazicMDNikicaGabricMD, PhD
