# Supplementary material for: Aflibercept With vs Without Reduced-Fluence Photodynamic Therapy for Polypoidal Choroidal Vasculopathy: A Randomized Clinical Trial
Source: JAMA Ophthalmol. 2025 Mar 27;143(5):393–9. doi: 10.1001/jamaophthalmol.2025.0250 (PMC11950976; doi:10.1001/jamaophthalmol.2025.0250)
Supplement: Supplement 3. — Data sharing statement [file jamaophthalmol-e250250-s003.pdf]

Data Sharing Statement

Chong. Aflibercept With vs Without Reduced-Fluence Photodynamic Therapy for Polypoidal Choroidal Vasculopathy. *JAMA Ophthalmol*. Published March 20, 2025.
doi:10.1001/jamaophthalmol.2025.0250

Data
Additional Information: NCT03941587
Data available: No

Additional Information
Explanation for why data not available: NCT03941587
